# Production of carotenoids from aromatics and pretreated lignocellulosic biomass by *Novosphingobium aromaticivorans*

**DOI:** 10.1128/aem.01268-23

**Published:** 2023-11-28

**Authors:** Benjamin W. Hall, Wayne S. Kontur, Jeanette C. Neri, Derek M. Gille, Daniel R. Noguera, Timothy J. Donohue

**Affiliations:** 1 DOE Great Lakes Bioenergy Research Center, University of Wisconsin, Madison, Wisconsin, USA; 2 Wisconsin Energy Institute, University of Wisconsin, Madison, Wisconsin, USA; 3 Laboratory of Genetics, University of Wisconsin, Madison, Wisconsin, USA; 4 Department of Civil and Environmental Engineering, University of Wisconsin, Madison, Wisconsin, USA; 5 Department of Bacteriology, University of Wisconsin, Madison, Wisconsin, USA; School of Life Sciences and Biotechnology, Shanghai, China

**Keywords:** carotenoids, coproducts, *Novosphingobium*, lignin, aromatics, PDC, nostoxanthin, CoQ_10_, astaxanthin, lycopene, β-carotene, zeaxanthin

## Abstract

Carotenoids are lipophilic compounds found in the membranes of various organisms. Individual carotenoids are also commodity chemicals, produced industrially for use as food additives, nutritional supplements, cosmetics, and pharmaceuticals. The alphaproteobacterium *Novosphingobium aromaticivorans* has previously been established as a potential platform microbe for converting aromatic compounds derived from lignocellulosic plant biomass into valuable extracellular products. Here, we show that *N. aromaticivorans* DSM 12444 cells naturally produce the carotenoid nostoxanthin, and we construct a set of gene deletion mutants that accumulate β-carotene, lycopene, or zeaxanthin, which are predicted intermediates in nostoxanthin biosynthesis as well as commodity chemicals. We also show that a mutant strain heterologously expressing a CrtW protein accumulates the carotenoid astaxanthin. When grown on vanillate as the carbon source, we find that the levels of carotenoids are not significantly affected by O_2_ concentration in the tested range of 5% to 21% O_2_. We also show that these carotenoids are produced at comparable levels when strains are grown in liquor from alkaline pretreated sorghum biomass [sorghum alkaline pretreatment liquor (APL)], which contains a mixture of aromatics. Finally, we construct strains that produce zeaxanthin, β-carotene, or astaxanthin concurrently with 2-pyrone-4,6-dicarboxylic acid, a potential building block for biodegradable polymers, when grown in sorghum APL. Combined, our results show that *N. aromaticivorans* can simultaneously produce valuable intracellular and extracellular commodities when grown in the presence of either pure aromatics or pretreated lignocellulosic biomass.

**IMPORTANCE:**

There is economic and environmental interest in generating commodity chemicals from renewable resources, such as lignocellulosic biomass, that can substitute for chemicals derived from fossil fuels. The bacterium *Novosphingobium aromaticivorans* is a promising microbial platform for producing commodity chemicals from lignocellulosic biomass because it can produce these from compounds in pretreated lignocellulosic biomass, which many industrial microbial catalysts cannot metabolize. Here, we show that *N. aromaticivorans* can be engineered to produce several valuable carotenoids. We also show that engineered *N. aromaticivorans* strains can produce these lipophilic chemicals concurrently with the extracellular commodity chemical 2-pyrone-4,6-dicarboxylic acid when grown in a complex liquor obtained from alkaline pretreated lignocellulosic biomass. Concurrent microbial production of valuable intra- and extracellular products can increase the economic value generated from the conversion of lignocellulosic biomass-derived compounds into commodity chemicals and facilitate the separation of water- and membrane-soluble products.

## INTRODUCTION

The aromatic polymer lignin is a major component of lignocellulosic plant biomass and is estimated to represent as much as 30% of the organic carbon in the biosphere (1). However, the heterogeneous structure and chemical composition of lignin have limited its economic value to industry. In addition, the mixture of aromatic compounds that results from lignocellulosic biomass deconstruction is often not metabolized by commonly used industrial microbes. We are interested in developing microbial catalysts that can convert heterogeneous mixtures of biomass-derived compounds, including aromatics, into valuable products.

We and others have been exploring *Novosphingobium aromaticivorans*, an alphaproteobacterium of the *Sphingomonadales* order, as a platform for producing valuable compounds (2) because it is amenable to genomic modification (3, 4) and can metabolize many components of deconstructed lignocellulosic biomass, including aromatic monomers (2) and some dimers (3, 5). For example, *N. aromaticivorans* DSM 12444 has been engineered to stoichiometrically convert the major aromatic monomers in deconstructed plant biomass into 2-pyrone-4,6-dicarboxylic acid (PDC), a potential polyester precursor (6) that is secreted into the media (2, 7). This study sought to expand the suite of valuable compounds that *N. aromaticivorans* can produce from biomass-derived aromatics.

Carotenoids are lipophilic isoprenoids that are produced by some plants, algae, bacteria, and fungi and function as membrane-bound light-harvesting pigments and antioxidants (8, 9). Several carotenoids (such as astaxanthin, β-carotene, lycopene, and zeaxanthin) are used industrially as animal feed, food coloring, nutritional supplements, cosmetics additives, and pharmaceuticals, with a 2017 global market size of ~$1.5B (10, 11). Most industrial carotenoids are produced synthetically (9, 10, 12), though there are a few biological sources commercially being used, such as the flower *Tagetes erecta* for lutein and the alga *Dunaliella salina* for β-carotene (10). Thus, there is growing interest in developing new biological sources of carotenoids (9, 10).

**Fig 1:**
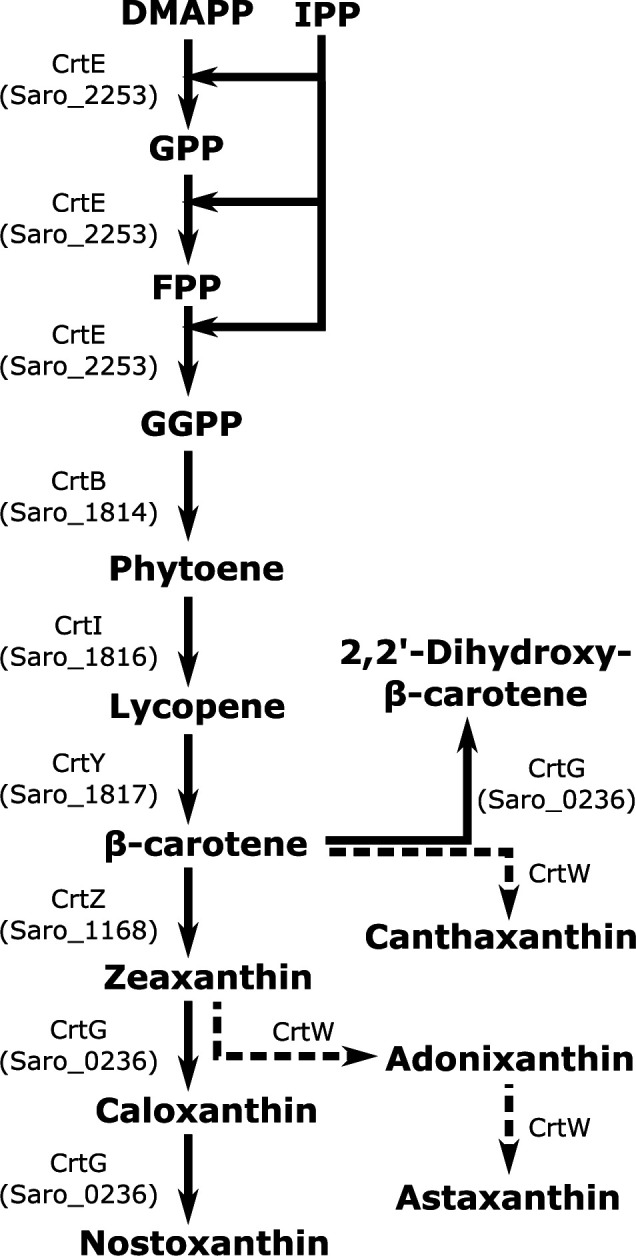
Predicted carotenoid biosynthetic pathway based on the annotated genome of *N. aromaticivorans*. Reactions predicted to be present in wild-type *N. aromaticivorans* (13, 15) are shown as full arrows and include the genes predicted to code for the enzymes involved. Reactions predicted to occur in strains in which *crtG* has been replaced in the genome with a *crtW* gene are shown as dashed arrows. DMAPP, dimethylallyl pyrophosphate; IPP, isopentenyl diphosphate; GPP, geranyl diphosphate; FPP, farnesyl diphosphate; and GGPP, geranylgeranyl pyrophosphate.

The genome sequence of *N. aromaticivorans* DSM 12444 predicts that this bacterium can produce the carotenoid nostoxanthin (13). Among the intermediates in the predicted nostoxanthin synthesis pathway of *N. aromaticivorans* are the industrially valuable carotenoids lycopene, β-carotene, and zeaxanthin (Fig. 1). A recent genome-scale metabolic model of *N. aromaticivorans* suggested that carotenoids could be some of the most profitable products made from plant biomass by *N. aromaticivorans* because of their high economic value and yields (14).

*N. aromaticivorans* (when it was known as *Sphingomonas aromaticivorans* F199) has also been shown to produce the lipophilic coenzyme Q_10_ (CoQ_10_) (16). CoQ_10_ is also the main isoprenoid quinone in humans and is a commodity chemical used in the pharmaceutical and cosmetics industries (17–20). Currently, bacteria that produce CoQ_10_ industrially (17, 21) cannot metabolize the aromatics present in deconstructed plant biomass. Thus, there is potential for *N. aromaticivorans* to also become a source of CoQ_10_ when grown in aromatic-containing solutions derived from plant biomass.

In this work, we test several predicted reactions in the *N. aromaticivorans* carotenoid biosynthetic pathway (Fig. 1) by generating defined mutants that accumulate β-carotene, lycopene, or zeaxanthin. Further, we engineer a strain that heterologously expresses a CrtW protein and accumulates the carotenoid astaxanthin. We show that these carotenoids can be produced from vanillate, an aromatic compound commonly present in deconstructed lignocellulosic plant biomass, and an alkaline pretreatment liquor (APL) made from sorghum. We also engineer a set of strains that produce either zeaxanthin, β-carotene, or astaxanthin concurrently with PDC when fed sorghum APL, showing that *N. aromaticivorans* can be engineered to simultaneously produce extracellular and intracellular products from this renewable carbon source. We discuss how the co-production of membrane-bound carotenoids and excreted dicarboxylic acids like PDC could improve the economics of valorizing biomass in a lignocellulosic biorefinery.

## RESULTS

### Nostoxanthin is the main carotenoid produced by *N. aromaticivorans* DSM 12444

**Fig 2:**
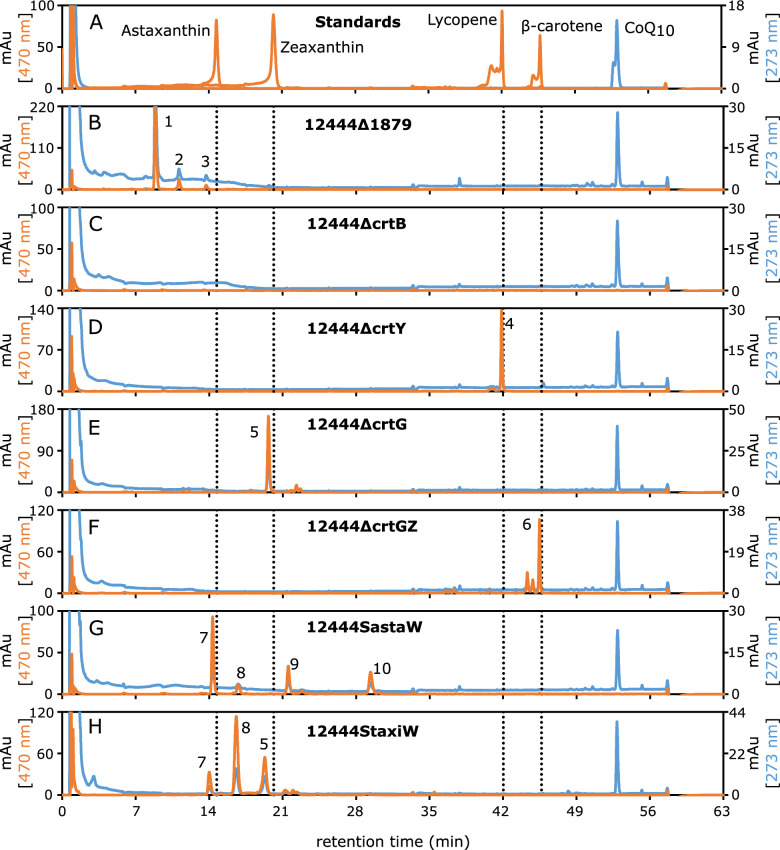
High-performance liquid chromatography (HPLC) analyses of acetone:methanol extracts of representative *N. aromaticivorans* cultures. Absorbances are shown for 273 nm (the wavelength of maximum absorbance for CoQ_10_; blue line) and 470 nm (a wavelength where all carotenoids investigated have some absorbance; orange line). Standard compounds (**A**) analyzed are astaxanthin, zeaxanthin, β-carotene, lycopene, and CoQ_10_; vertical dotted lines in panels B–H show the time of maximum absorbance for the carotenoid standards. The strains analyzed are 12444Δ1879 (**B**), 12444ΔcrtB (**C**), 12444ΔcrtY (**D**), 12444ΔcrtG (**E**), 12444ΔcrtGZ (**F**), 12444SastaW (**G**), and 12444StaxiW (**H**). Data are shown as milliabsorbance units (mAu). Major carotenoid peaks are numbered from 1 to 13. Absorbance spectra for all major peaks are shown in Fig. S1 to S8.

The *N. aromaticivorans* DSM 12444 genome predicts that this bacterium contains genes that encode previously uncharacterized proteins with 52% to 74% amino acid sequence identity to known enzymes that lead to nostoxanthin production (Table S1). To test the prediction that *N. aromaticivorans* uses these previously uncharacterized gene products to produce carotenoids, we grew cells in the presence of vanillate and analyzed acetone:methanol (lipophilic) extracts of the cells by liquid chromatography mass spectrometry (LC-MS). Our analysis of these extracts from the parent *N. aromaticivorans* strain (12444Δ1879) was consistent with nostoxanthin being a major carotenoid (compound 1 in Fig. 2B): compound 1 had absorbance maxima at 453 and 480 nm (Fig. S2B) and an m/z peak of 600 (Fig. S2C), both characteristic of nostoxanthin (22, 23). Two other compounds in these extracts had slightly longer retention times than compound 1: compound 2 had absorbance maxima at 339, 446, and 474 nm (Fig. S2B) and m/z peaks of 600 and 639 (Fig. S2C), and compound 3 had absorbance maxima at 453 and 481 nm (Fig. S2B) and an m/z peak of 584 (Fig. S2C). Although we were not able to identify compound 2, the properties of compound 3 are consistent with caloxanthin (23), a predicted precursor to nostoxanthin (Fig. 1) known to be present in some sphingomonads that produce nostoxanthin (15, 23). However, commercial standards of pure nostoxanthin or caloxanthin were not available to estimate the compounds’ abundances or provide further proof of their chemical identity. Another compound detected in the *N. aromaticivorans* lipophilic extracts had the same retention time and absorbance spectrum as a commercial CoQ_10_ standard (Fig. 2A and B; Fig. S1 and S2B), consistent with previous work showing that CoQ_10_ is the major quinone in this bacterium (16).

### Lipophilic compounds produced by *N. aromaticivorans* mutants containing deletions of genes in the predicted carotenoid biosynthetic pathway

To further test whether *N. aromaticivorans* uses the predicted carotenoid biosynthesis pathway (Fig. 1), we generated a set of mutants with in-frame deletions of genes predicted to encode proteins involved in the pathway. We grew these strains with vanillate as the sole carbon source and analyzed the compounds present in lipophilic extracts from these mutants, using commercial standards when available to aid in the identification and quantification of the lipophilic compounds.

**Fig 3:**
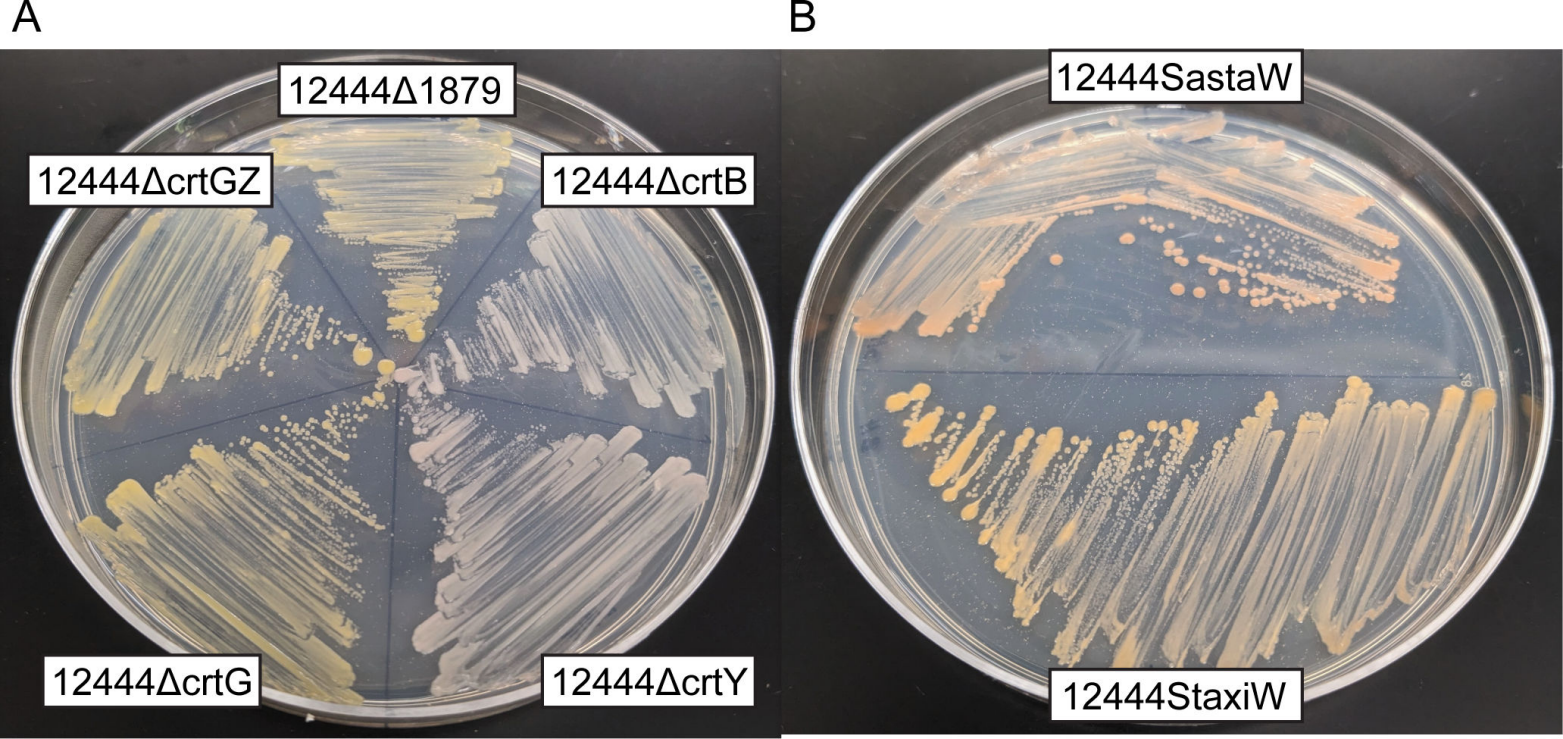
Standard mineral base (SMB)-glucose plates of parent and mutant *N. aromaticivorans* strains showing the different colony colors in carotenoid pathway deletion mutants (**A**) or in *crtW*-expressing engineered strains (**B**). Some colony colors appear similar despite accumulating different carotenoids (Fig. 2) due to the known similarity in the absorption spectra of their carotenoids (Fig. S1 to S8).

Deletion of Saro_1814 (encoding a putative CrtB homolog) resulted in a strain (12444ΔcrtB) that formed nonpigmented colonies on solid media (Fig. 3A), as expected given the predicted role of this gene product in phytoene synthesis (Fig. 1). We also found that strain 12444ΔcrtB only contained CoQ_10_ as a major lipophilic compound (Fig. 2C; Fig. S3). Deletion of Saro_1817 (encoding a putative CrtY homolog) resulted in a strain (12444ΔcrtY) that formed light pink colonies (Fig. 3A) and contained the predicted pathway intermediate lycopene (compound 4) and CoQ_10_ in its lipophilic extract (Fig. 2D; Fig. S4). Deletion of Saro_0236 (encoding a putative CrtG homolog) resulted in a strain (12444ΔcrtG) that formed yellow colonies (Fig. 3A) and contained the predicted pathway intermediate zeaxanthin (compound 5) and CoQ_10_ as the major components of its lipophilic extract (Fig. 2E; Fig. S5). Finally, to test if the pathway intermediate β-carotene could be accumulated, we constructed a strain (12444ΔcrtGZ) in which both Saro_0236 and Saro_1168 (encoding a putative CrtZ homolog were deleted, as deletion of Saro_1168 alone would be expected to also produce 2,2ʹ-dihydroxy-β-carotene (Fig. 1). Strain 12444ΔcrtGZ formed yellow colonies (Fig. 3A), and the main components of its lipophilic extract were β-carotene (compound 6) and CoQ_10_ (Fig. 2F; Fig. S6).

In sum, the compounds present in the lipophilic extracts of each of these mutants were consistent with predictions from the annotated carotenoid biosynthetic pathway in the *N. aromaticivorans* genome (Fig. 1). These experiments illustrate that one or more gene deletions can result in *N. aromaticivorans* strains that produce industrially valuable carotenoids when grown in the presence of vanillate. Furthermore, the lipophilic extracts from all strains tested also contain the electron carrier CoQ_10_.

### Production of astaxanthin by an engineered *N. aromaticivorans* strain

Astaxanthin is a valuable carotenoid that is not predicted to be produced by *N. aromaticivorans*, since the genome of this organism lacks a *crtW* gene. While several bacteria, including some other members of the *Sphingomonadales* order [*Sphingomonas astaxanthinifaciens* (24) and *Sphingomonas taxi* ATCC 55669 (25)], naturally produce astaxanthin (26), none of these are known to metabolize aromatic compounds present in pretreated lignocellulosic biomass. To test if we could engineer *N. aromaticivorans* to produce astaxanthin, we placed a recombinant *crtW* gene from *S. astaxanthinifaciens* or *S. taxi* separately into the *crtG* locus of 12444ΔcrtG to generate strains 12444SastaW and 12444StaxiW, respectively. The difference in colony colors of the 12444ΔcrtG, 12444SastaW, and 12444StaxiW strains (Fig. 3) suggested that the insertion of each of these *crtW* genes into the *N. aromaticivorans* genome resulted in altered carotenoid profiles.

To test this hypothesis, we analyzed the lipophilic extracts from vanillate-grown 12444SastaW and 12444StaxiW cells. This analysis showed that 12444SastaW produced astaxanthin (Fig. 2G; compound 7), as well as small amounts of three other putative carotenoids (compounds 8, 9, 10) and CoQ_10_ (Fig. 2G; Fig. S7). The lipophilic extract of 12444StaxiW also contained astaxanthin (compound 7) and CoQ_10_ (Fig. 2H; Fig. S8), although its predominant carotenoid (compound 8, which is also a minor component of the 12444SastaW extract) appears to be adonixanthin, based on its measured mass (m/z peak = 583; Fig. S8C). The 12444StaxiW lipophilic extract also contained two additional compounds, one of them identified as zeaxanthin (compound 5; Fig. 2H; Fig. S8). From this, we conclude that introducing the CrtW protein from *S. astaxanthinifaciens* into *N. aromaticivorans* generates a strain that is more effective at accumulating astaxanthin than a strain using the CrtW protein from *S. taxi*. Therefore, in subsequent experiments, we used cells containing the *S. astaxanthinifaciens crtW* gene (strain 12444SastaW) as a chassis for an astaxanthin-producing strain of *N. aromaticivorans*.

### Impact of O_2_ tension on levels of carotenoids and CoQ_10_ in *N. aromaticivorans*

The dissolved O_2_ concentration of a culture can affect carotenoid levels in various organisms in different ways (27). For example, lower O_2_ tensions have been shown to increase CoQ_10_ production in some bacteria (21), while other microbes increase carotenoid production at high O_2_ tensions presumably since carotenoids can provide protection against reactive oxygen species (27). Therefore, we tested whether bubbling *N. aromaticivorans* cultures with a gas containing 5%, 10%, or 21% O_2_ would lead to significant changes in carotenoids and CoQ_10_ levels when using vanillate as a carbon source.

**Fig 4:**
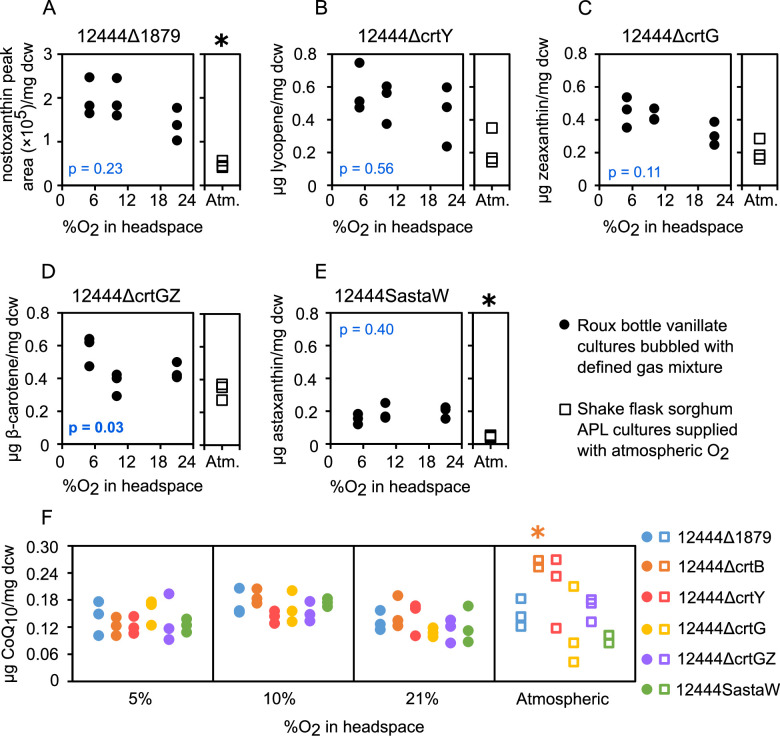
Carotenoid and CoQ_10_ levels in *N. aromaticivorans* strains. The filled circles show data for roux bottle vanillate-fed cultures bubbled with a gas mixture of defined composition, and the open squares show data for shake flask sorghum APL cultures supplied with atmospheric O_2_. The amounts of select carotenoids, normalized by dcw, are plotted for *N. aromaticivorans* strains 12444Δ1879 producing nostoxanthin (**A**), 12444ΔcrtY producing lycopene (**B**), 12444ΔcrtG producing zeaxanthin (**C**), 12444ΔcrtGZ producing β-carotene (**D**), and 12444SastaW producing astaxanthin (**E**). Data for roux bottle vanillate cultures were plotted against headspace O_2_ concentration, and *P*-values are shown for single-factor ANOVA tests (*α* = 0.05) comparing the data at all three O_2_ concentrations for each strain (null hypothesis assuming no difference at the three O_2_ concentrations). CoQ_10_ levels are also shown for those same strains, plus 12444ΔcrtB, grown in roux bottles with vanillate and shake flasks with sorghum APL (**F**). Nostoxanthin accumulation is reported as an HPLC peak area due to the lack of a standard. For all other compounds, the amounts are reported as mass normalized to dry cell weight. An asterisk (*) denotes data from shake flask sorghum APL cultures that are significantly different from 21% O_2_ roux bottle culture data (Student’s *t*-test, *P* < 0.05).

Carotenoid levels of *N. aromaticivorans* strains grown at different O_2_ tensions are shown in Fig. 4A through E. Nostoxanthin levels in 12444Δ1879, which are reported as HPLC peak area due to lack of a standard for quantification, showed only modest changes with oxygen [ranging from 1.0 to 2.5 × 10^5^ area units per mg dry cell weight (dcw)]. Under the same growth conditions, lycopene levels in 12444ΔcrtY ranged from 0.24 to 0.75 µg/mg dcw, zeaxanthin in 12444ΔcrtG ranged from 0.25 to 0.54 µg/mg dcw, β-carotene in 12444ΔcrtGZ ranged from 0.29 to 0.64 µg/mg dcw, and astaxanthin in 12444SastaW ranged from 0.12 to 0.25 µg/mg dcw. Single-factor ANOVA tests (alpha = 0.05) of carotenoid levels suggested that only strain 12444ΔcrtGZ contained significantly different carotenoid levels at the O_2_ concentrations tested (Fig. 4A through E). CoQ_10_ levels for each strain at different O_2_ tensions (Fig. 4F) ranged from 0.08 to 0.2 µg/mg dcw. ANOVA tests also showed that, for each of the strains, there was no significant difference in CoQ_10_ production at the three O_2_ concentrations tested (Fig. 4F). Furthermore, ANOVA tests showed there was no significant difference in CoQ_10_ production between the different strains at any of the individual O_2_ concentrations. Therefore, we conclude that O_2_ availability does not have a consistently significant impact on *N. aromaticivorans* carotenoid or CoQ_10_ levels over the concentration range tested.

### Production of carotenoids and CoQ_10_ by *N. aromaticivorans* from alkaline pretreated sorghum biomass

The above results showed that wild-type and engineered *N. aromaticivorans* strains accumulate carotenoids and CoQ_10_ when grown on vanillate, an aromatic compound predicted to be found in deconstructed lignocellulosic biomass. However, we also wanted to test whether carotenoids and CoQ_10_ could be generated when cells were grown on the mixture of aromatics directly obtained from plant biomass. We therefore grew several strains in a sorghum APL (28), which contains a mixture of aromatic monomers and other organics (Fig. S9; Table S2). With a few exceptions, the *N. aromaticivorans* strains grown in sorghum APL (in shake flasks with atmospheric O_2_ conditions) produced amounts of carotenoids and CoQ_10_ (normalized by dry cell weight; open squares in Fig. 4) that were within the production ranges of the vanillate-grown cultures grown at different dissolved O_2_ tensions (filled circles in Fig. 4). One exception was strain 12444ΔcrtB, which lacks detectable carotenoids in lipophilic extracts; this strain produced significantly more CoQ_10_ when grown in APL compared to when grown on vanillate (Fig. 4F). In addition, strains 12444Δ1879 and 12444SastaW produced significantly less nostoxanthin and astaxanthin, respectively, when grown in APL compared to when grown on vanillate. Overall, these results show that it is possible to use *N. aromaticivorans* for producing carotenoids from a mixture of aromatic and other organic compounds derived from pretreated lignocellulosic biomass.

### Concurrent production of carotenoids, CoQ_10_, and PDC from alkaline pretreated sorghum biomass

**Fig 5:**
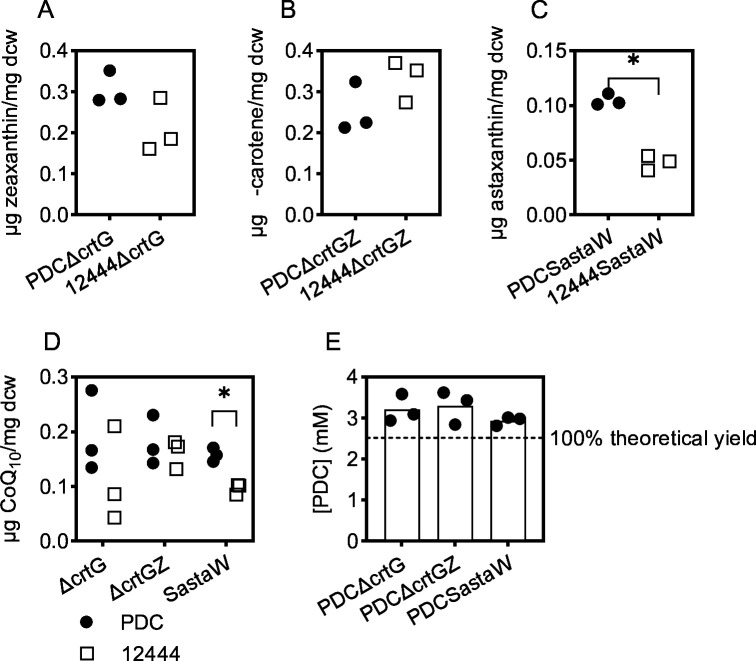
Carotenoid (**A–C**), CoQ_10_ (**D**), and PDC (**E**) levels in *N. aromaticivorans* PDC strains grown in sorghum APL. Filled circles show data for PDC-producing strains, open squares show data for non-PDC-producing strains grown in sorghum APL reproduced from Fig. 4. The amounts of carotenoids and are reported as mass normalized to dcw. PDC levels are reported as concentration measured in the media. An asterisk (*) denotes PDC strain data that are significantly different from the 12444 parent strain data (Student’s *t*-test, *P* < 0.05).

Previous work has shown that engineered *N. aromaticivorans* strains containing defined mutations in aromatic metabolism can convert the three major classes of biomass aromatics (syringyl, guaiacyl, and *p*-hydroxyphenyl) into PDC and secrete it into the medium (2, 7). We sought to test whether individual *N. aromaticivorans* strains could produce both extracellular PDC and intracellular lipophilic compounds (carotenoids and CoQ_10_) as valuable products from biomass-derived media. To do this, we generated a set of strains that contained both the mutations needed to accumulate extracellular PDC and those needed to accumulate the carotenoids zeaxanthin, β-carotene, or astaxanthin. We found that these engineered strains produce extracellular PDC, as well as the expected carotenoid and CoQ_10_ when grown in sorghum APL (Fig. 5). The levels of individual carotenoid species and CoQ_10_ (normalized by dry cell weight) in the lipophilic cell extracts are comparable to or greater than the amounts produced by the respective non-PDC-producing strains grown in sorghum APL (Fig. 5A through D). In addition, the levels of extracellular PDC are equal to or greater than 100% theoretical yield, based on the measured concentrations of aromatic compounds in APL (Fig. 5E; Table S2). This observation is consistent with the stoichiometric conversion of aromatic monomers to PDC reported previously for the PDC-producing strain that was used in these studies (7).

## DISCUSSION

This work sought to expand the types of valuable chemicals that could be produced from pretreated lignocellulosic biomass. We confirmed that *N. aromaticivorans* naturally produces the industrially important isoprenoid CoQ_10_, and we leveraged its native ability to synthesize carotenoids along with the utility of heterologous expression to engineer mutant strains that accumulate different valuable carotenoids from either a pure aromatic (vanillate) or from sorghum APL, a feedstock derived from lignocellulosic biomass. We also used this new information to engineer a set of *N. aromaticivorans* strains that can concurrently produce CoQ_10_, a valuable carotenoid, and PDC from sorghum APL.

### *N. aromaticivorans* as a production platform for carotenoids and CoQ_10_

Although other microbes can be used as sources of carotenoids and CoQ_10_, our work is important for several reasons. First, we confirmed predictions from *N. aromaticivorans* genomic and physiological studies that this bacterium contains metabolic pathways that can produce the carotenoid nostoxanthin (13) as well as CoQ_10_ (16). Our work also shows that minimal genomic modifications of *N. aromaticivorans* can lead to strains that accumulate valuable carotenoids such as β-carotene, lycopene, astaxanthin, or zeaxanthin. In addition, we demonstrate that *N. aromaticivorans* cells lacking *crtB* (Saro_1814) could simplify industrial production of CoQ_10_, another important commodity chemical used in the pharmaceutical and cosmetics industries, because the *crtB* mutation prevents the accumulation of other acetone:methanol soluble materials.

We also found that changes in O_2_ tension did not have a significant impact on *N. aromaticivorans* carotenoid or CoQ_10_ levels, unlike in other microbes where O_2_ tensions or the resulting oxidative stress can have a significant impact on the accumulation of these products (27). Effects of O_2_ tension on carotenoid production can be variable; in some cases, lower O_2_ tensions lead to higher production due to increased membrane synthesis (29), while in other cases, higher O_2_ tensions lead to higher production (30) presumably due to the ability of these compounds to quench reactive oxygen species (27). Although our data suggests that *N. aromaticivorans* might not regulate carotenoid synthesis in response to changes in O_2_ tension, additional studies are needed to test this hypothesis. The ability of *N. aromaticivorans* to synthesize comparable levels of carotenoids and CoQ_10_ at different O_2_ concentrations could be advantageous in industrial settings, due to the capital and operational costs associated with the aeration of large bioreactors.

### Microbial production of valuable commodity chemicals from pretreated lignocellulosic biomass

As society looks for ways to produce commodity chemicals from abundant renewable resources, pretreated lignocellulosic biomass is an attractive material. *N. aromaticivorans* has natural converging pathways to catabolize major components found in pretreated lignocellulosic biomass, including the most abundant aromatic monomers found in lignin (syringyl, guaiacyl, and p-hydroxyphenyl aromatics) (2), some aromatic dimers (3, 5), and other organic compounds (31). Because carotenoids and CoQ_10_ are produced from central metabolites, *N. aromaticivorans* could thus “funnel” mixtures of compounds found in pretreated lignocellulosic biomass into these commodity chemicals. This is in contrast to existing microbial hosts for producing carotenoids or CoQ_10_ that typically use food-grade sugars as carbon sources (32). To date, relatively few of the microbes being considered for industrially producing carotenoids and CoQ_10_ also have the native ability to metabolize any aromatic compounds (33–37). Thus, *N. aromaticivorans* could be an important microbial catalyst for the industrial production of carotenoids and CoQ_10_ from renewable lignocellulosic carbon sources.

The method used to generate the sorghum APL feedstock is well known to solubilize easily cleavable aromatics from plant cell walls, without the breakdown of the carbohydrate and lignin polymers in the biomass (28). We showed that *N. aromaticivorans* can grow in the presence of sorghum APL alone, unlike previous studies which have supplemented lignocellulosic APL with minerals (38–43) and/or nitrogen/carbon sources (38, 40, 44, 45). We also found that *N. aromaticivorans* produces nearly the same amounts (normalized to dry cell weight) of carotenoids and CoQ_10_ when grown on sorghum APL as when grown in a defined medium containing vanillate (Fig. 4), in addition to accumulating comparable amounts of cellular material (Fig. S10). These results suggest that *N. aromaticivorans* could produce these valuable isoprenoids from lignocellulosic substrates without the need to add other nutrients.

### Simultaneous production of carotenoids, CoQ_10_, and PDC from pretreated lignocellulosic biomass

Our work illustrates the potential for *N. aromaticivorans* to produce carotenoids and CoQ_10_ as intracellular lipophilic products. We also generated strains that concurrently produce these lipophilic products along with the soluble extracellular product PDC, which has several potential industrial uses (46). In strains that accumulate both intracellular (carotenoids and CoQ_10_) and extracellular (PDC) products, the cellular and aqueous fractions can be separated and each used as a source of valuable products to increase the economic value derived from pretreated lignocellulosic biomass. Notably, the strains we engineered for simultaneous PDC and carotenoid production accumulate at least as much carotenoid as the non-PDC-producing strains, showing that the synthesis of two products does not have a significant negative impact on the overall output. The greater than 100% theoretical yield of PDC observed for these strains (Fig. 5E) likely reflects the conversion of aromatic compounds present in the sorghum APL that were not detected by our HPLC-MS/MS analysis, in addition to the complete conversion of detected aromatic monomers (Table S2).

In previous studies with strains that produce PDC from aromatics, a second carbon source (glucose) was added to cells, since the mutations that result in accumulation of PDC block the use of aromatics to support growth (2, 7). The growth of PDC-producing strains in sorghum APL reported here predicts that *N. aromaticivorans* will not need to be supplemented with other nutrients to produce extracellular and intracellular compounds from this and possibly other types of feedstocks derived from plant biomass.

In sum, this work adds to a growing body of evidence that *N. aromaticivorans* is a promising microbe for converting lignocellulosic biomass into valuable compounds because it is amenable to genomic modifications and can metabolize abundant aromatic components of this biomass. This work establishes *N. aromaticivorans* as a promising host for producing valuable carotenoids and CoQ_10_ from both pretreated lignocellulosic biomass and purified aromatics. In addition, *N. aromaticivorans* has the ability to produce these intracellular lipophilic compounds concurrently with PDC, which could help to improve the economics of converting plant biomass into industrial commodities. Future work will focus on improving yields of these and other products under industrially relevant conditions.

## MATERIALS AND METHODS

### *Novosphingobium aromaticivorans* strains

Details on all strains in this study can be found in Table 1. *N. aromaticivorans* 12444Δ1879 is a derivative of wild-type strain DSM 12444 (also called F199 [31, 47]), in which a putative *sacB* gene (Saro_1879 or SARO_RS09410) was deleted to create a strain amenable to genomic modifications using a *sacB*-containing plasmid (3, 48). We used 12444Δ1879 as the parent strain to generate strains 12444ΔcrtB (lacking *crtB*; Saro_1814 or SARO_RS09080), 12444ΔcrtY (lacking *crtY*; Saro_1817 or SARO_RS09095), 12444ΔcrtG (lacking *crtG*; Saro_0236 or SARO_RS01180), 12444ΔcrtGZ (lacking *crtG* and *crtZ*; Saro_0236 and Saro_1168 or SARO_RS01180 and SARO_RS05825), 12444StaxiW [replacing Saro_0236 with the gene for the CrtW protein from *S. taxi* ATCC 55669 (NCBI accession WP_038660513.1)], and 12444SastaW (replacing Saro_0236 with the gene for the CrtW protein from *S. astaxanthinifaciens* [NCBI accession WP_211248127.1)].

**TABLE 1:** All strains and plasmids used in this study

Name	Genotype	Description	Reference
*Escherichia coli* strains			
DH5α	F- Φ80lacZΔM15 Δ(lacZYA-argF) U169 recA1 endA1 hsdR17 (rK-,mk+) phoA supE44 λ-thi- gyrA96 relA1	Used for creating and maintaining plasmids	Bethesda Research Laboratories
S17-1	recA pro hsdR RP4-2-Tc::Mu-Km::Tn7	Used for mobilizing plasmids into *N. aromaticivorans* via conjugation	(49)
*N. aromaticivorans* strains			
12444Δ1879	DSM 12444 ΔSaro_1879	Parent strain; putative *sacB* has been deleted to allow genomic modifications using a *sacB*-containing plasmid	(3)
12444ΔcrtB	DSM 12444 ΔSaro_1879 ΔSaro_1814	Parent with deleted *crtB*	This work
12444ΔcrtY	DSM 12444 ΔSaro_1879 ΔSaro_1817	Parent with deleted *crtY*	This work
12444ΔcrtG	DSM 12444 ΔSaro_1879 ΔSaro_0236	Parent with deleted *crtG*	This work
12444ΔcrtGZ	DSM 12444 ΔSaro_1879 ΔSaro_0236 ΔSaro_1168	Parent with deleted *crtG* and *crtZ*	This work
12444StaxiW	DSM 12444 ΔSaro_1879 ΔSaro_0236::crtW from *S. taxi* ATCC 55669	Parent with deleted *crtG*, with the gene for the CrtW protein from *Sphingomonas taxi* ATCC 55669 (NCBI Accession WP_038660513.1) in the Saro_0236 genomic locus	This work
12444SastaW	DSM 12444 ΔSaro_1879 ΔSaro_0236::crtW from *S. astaxanthinifaciens*	Parent with deleted *crtG*, with the gene for the CrtW protein from *Sphingomonas astaxanthinifaciens* (NCBI accession WP_211248127.1) in the Saro_0236 genomic locus	This work
12444PDCΔ*dmtS*	DSM 12444 ΔSaro_1879 ΔSaro_2819 ΔSaro_2864-5 ΔSaro_1872	Parent with deleted *ligI*, *desCD*, and *dmtS* that accumulates PDC from aromatic monomers	(7)
PDCΔcrtG	DSM 12444 ΔSaro_1879 ΔSaro_2819 ΔSaro_2864-5 ΔSaro_1872 ΔSaro_0236	12444PDCΔ*dmtS* with deleted *crtG*	This work
PDCΔcrtGZ	DSM 12444 ΔSaro_1879 ΔSaro_2819 ΔSaro_2864-5 ΔSaro_1872 ΔSaro_0236 ΔSaro_1168	12444PDCΔ*dmtS* with deleted *crtG* and *crtZ*	This work
PDCSastaW	DSM 12444 ΔSaro_1879 ΔSaro_2819 ΔSaro_2864-5 ΔSaro_1872 ΔSaro_0236::crtW from *S. axtaxanthinifaciens*	12444PDCΔ*dmtS* with deleted *crtG*, with the gene for the CrtW protein from *S. astaxanthinifaciens* in the Saro_0236 genomic locus	This work
Plasmids			
pK18msB-MCS1		pK18*mobsacB* lacking the multiple cloning site, with a new XbaI site introduced	(3, 48)
pK18msB/ΔSaro1814		pK18msB-MCS1 containing genomic regions that naturally flank Saro_1814	This work
pK18msB/ΔSaro1817		pK18msB-MCS1 containing genomic regions that naturally flank Saro_1817	This work
pK18msB/ΔSaro0236		pK18msB-MCS1 containing genomic regions that naturally flank Saro_0236	This work
pK18msB/ΔSaro1168		pK18msB-MCS1 containing genomic regions that naturally flank Saro_1168	This work
pK18msB/ΔSaro0236::StaxiW		pK18msB/ΔSaro0236 with the gene for CrtW from *S. taxi* ATCC 55669 between the Saro_0236 flanking regions	This work
pK18msB/ΔSaro0236::SastaW		pK18msB/ΔSaro0236 with the gene for CrtW from *S. astaxanthinifaciens* between the Saro_0236 flanking regions	This work

*N. aromaticivorans* 12444PDCΔ*dmtS* is a derivative of 12444Δ1879 that was genetically modified to accumulate stoichiometric amounts of PDC from syringyl, guaiacyl, and *p*-hydroxyphenyl aromatic compounds (7). The strain 12444PDCΔ*dmtS* has Saro_2819 (*ligI*), Saro_2864–5 (*desCD*), and Saro_1872 (*dmtS*) deleted from the genome. We used 12444PDCΔ*dmtS* as the parent strain to generate strains PDCΔ*crtG* (lacking Saro_0236), PDCΔcrtGZ (lacking both Saro_0236 and Saro_1168), and PDCSastaW (replacing Saro_0236 with the gene for the CrtW protein from *S. astaxanthinifaciens*).

Genes for CrtW proteins were synthesized as gBlocks (Integrated DNA Technologies, Coralville, IA, USA). Plasmids for cloning were constructed with the NEBuilder HiFi DNA Assembly Master Mix (New England Biolabs, Ipswich, MA, USA). Methods for constructing mutants [including PCR primers used (Table S5)] are contained in Supplementary Information.

### Bacterial growth

*E. coli* strains used for plasmid cloning were grown in lysogeny broth and shaken at ~200 rpm at 30°C or 37°C. For routine manipulation, *N. aromaticivorans* cultures were grown in GluSis at 30°C. GluSis is a modification of Sistrom’s minimal medium (50) in which the succinate has been replaced by 22.6-mM glucose. The minimal medium used for *N. aromaticivorans* experiments was SMB (3) at an initial pH of 7.0. Where needed to select for the presence or absence of plasmids, media were supplemented with 100-µg/mL ampicillin, 50-µg/mL kanamycin, or 10% sucrose (wt/vol).

### Preparation of sorghum APL

Sorghum APL (45) was prepared by mixing samples of milled 2014 GLBRC sorghum (2 g) with a sodium hydroxide solution (1% NaOH in H_2_O, 20 mL) in sealed 125-mL Erlenmeyer flasks, before heating for 90 min in an oil bath at 90°C. The flask was then immediately placed in ice for 10 min, after which the biomass and aqueous phases were separated by centrifugation at 4,300 × *g* for 15 min and the supernatant was recovered as a source of soluble aromatics. The solid biomass was rinsed three times with ddH_2_O (20 mL, 15 mL, and 15 mL), and the washes were recovered through centrifugation. The initial aqueous supernatant and washes were combined and adjusted to pH 7.0 using 1-M HCl. The solution was centrifuged at 20,000 × *g* for 1 h at 4°C and passed through a 0.2-µM surfactant-free cellulose acetate (SFCA) filter to remove any remaining insoluble material, yielding the APL used in further experiments.

### Growth of *N. aromaticivorans* in minimal medium with vanillate

Cultures of each *N. aromaticivorans* strain were initially grown in a 125-mL conical shake flask containing 10-mL SMB supplemented with 4-mM vanillate. Between 3 and 8 mL of this culture was combined with 480 mL of fresh SMB + 4-mM vanillate in a glass roux bottle. Roux bottle cultures were attached to a gas mixer using SideTrak 840 mass flow controllers attached to a FloBox 954 (Sierra Instruments, Monterey, CA, USA) in a 30°C temperature-controlled room. Gas was piped into the bottoms of the cultures and exhausted from the headspace through outlets in stoppers. The gas contained 5, 10, or 21% O_2_, 1% CO_2_, and N_2_ as the remainder. Cell growth was monitored by periodically removing samples for analysis using a Klett-Summerson photoelectric colorimeter with a red filter. Cultures were grown until they reached late exponential growth or early stationary phase. For dcw determination, aliquots (~80 mL) were centrifuged in pre-weighed tubes (8,000 × *g* for 15 min), supernatants were removed, cell pellets were air-dried in a fume hood, and then the tubes were reweighed (Fig. S10). Aliquots (~160 mL) were also harvested (centrifuged at 8,000 × *g* for 15 min) for isolation of lipophilic compounds by extraction with acetone:methanol (see below).

### Growth of *N. aromaticivorans* in sorghum APL

Each *N. aromaticivorans* strain was initially grown in a 125-mL conical shake flask containing 10-mL SMB supplemented with 10-mM glucose. In addition, 1-mL aliquots were centrifuged at ~7,000 × *g* for 5 min, the supernatant was removed, and the cell pellet was used to inoculate 18 mL of sorghum APL in a 125-mL conical shake flask. Cultures were shaken at ~200 rpm at 30°C until they reached the early stationary phase. Aliquots of cultures for extraction into acetone:methanol (10 mL) and dcw determination (5 mL) were harvested as described above (see Fig. S10 for dry cell weight measurements).

### Preparation of lipophilic extracts

Care was taken to minimize O_2_ and light exposure to acetone:methanol extracts, although samples were not handled anaerobically. Cell pellets were resuspended in water (950 µL for roux bottle samples and 100 µL for pellets from shake flask cultures) and then transferred into a 15-mL glass Sorvall centrifuge tubes. Extraction solvent (7:2 acetone:methanol solution; 5 mL or 1.5 mL, respectively, for roux bottle or shake flask samples) was added, and the samples were mixed by pipetting. The tube was centrifuged (10,000 × *g* for 20 min), and then the supernatant was transferred to a new 15 mL glass tube. The pelleted cells were extracted a second time, after resuspending cells in water (500 µL or 100 µL, respectively, for roux bottle or shake flask samples) followed by extraction solvent (4.5 mL or 1.5 mL, respectively, for roux bottle or shake flask samples). After centrifugation, the supernatants from both extractions were combined. The combined supernatants were partially dried under a stream of N_2_ (to a final volume of ~1–4 mL) to concentrate materials before analysis by HPLC. The concentration of compounds in lipophilic extracts was calculated after correcting for dry cell weight, any dilution prior to extraction, and the final volume of the sample after drying under N_2_.

### HPLC identification and quantification of lipophilic compounds

For identification and quantification, the acetone:methanol lipophilic extracts were analyzed via reverse-phase HPLC using a Kinetex 2.6-µM PS C18 100 Å (150 × 2.1 mm) column (Phenomenex, Torrance, CA, USA) attached to a Shimadzu Nexera XR HPLC system. The mobile phase was a binary gradient (Fig. S11) of Solvent A (70% acetonitrile/30% water) and Solvent B (70% acetonitrile/30% isopropanol) flowing at 0.45 mL/min. Absorbance was measured between 200 and 600 nm using a Shimadzu SPD-M20A photodiode array detector. The following commercial standards were used to identify compounds in the lipophilic extracts: β-carotene (Sigma-Aldrich), lycopene [Pharmaceutical Secondary Standard, Certified Reference Material (7.2%), Supelco], zeaxanthin (United States Pharmacopeia Reference Standard), astaxanthin (Sigma-Aldrich), and coenzyme Q_10_ (Sigma-Aldrich).

To identify compounds that were not commercially available for use as standards, the eluent from the HPLC was analyzed via mass spectrometry using a Shimadzu triple quadrupole mass spectrometer LCMS-8045. We used positive mode Q3 scans from 450 m/z to 700 m/z around the retention times of unknown HPLC peaks to obtain mass spectra of compounds eluting at such times (Fig. S2 and S8).

### Analysis of culture media for PDC and aromatic compounds

Extracellular media samples were prepared by centrifuging 1.5 mL of culture at 20,000 × *g* for 2 min before passing the supernatant through a 0.2-µM SFCA membrane filter. The filtered media was analyzed using the Shimadzu Nexera XR HPLC system with the photodiode array detector and LCMS-8045 described above. The mobile phase was a binary gradient (Fig. S12) of Solvent A (0.2% formic acid in water) and Solvent B (methanol) flowing at 0.4 mL/min. The stationary phase was a Phenomenex Kinetex F5 column (2.6-µM pore size, 2.1-mm ID, 150-mm length). Aromatic compounds were identified by multiple-reaction monitoring (MRM) using the transition ions specified in Table S3, which were obtained from analyzing pure standards as previously described (7). Aromatic compounds were quantified by comparing sample absorbance at specific wavelengths and retention times with known standards, as measured by the photodiode array detector (Table S4).

## SUPPLEMENTAL MATERIAL

Supplementary Information aem.01268-23-s0001.docx Supplementary text, figures, and tables.

ASM does not own the copyrights to Supplemental Material that may be linked to, or accessed through, an article. The authors have granted ASM a non-exclusive, world-wide license to publish the Supplemental Material files. Please contact the corresponding author directly for reuse.
