# Supplementary material for: Production of carotenoids from aromatics and pretreated lignocellulosic biomass by Novosphingobium aromaticivorans
Source: Appl Environ Microbiol. 2023 Nov 28;89(12):e01268-23. doi: 10.1128/aem.01268-23 (PMC10734531; doi:10.1128/aem.01268-23)
Supplement: Supplementary Information — Supplementary text, figures, and tables. [file aem.01268-23-s0001.docx]

**Electronic Supplementary Information**

# Production of carotenoids from aromatics and pretreated lignocellulosic biomass by *Novosphingobium aromaticivorans*

Benjamin W. Hall,^a,b,c‡^ Wayne S. Kontur,^a,b‡^ Jeanette C. Neri,^a,b^* Derek M. Gille,^a,b^* Daniel R. Noguera,^a,b,d^ Timothy J. Donohue^a,b,e^#

*^a^DOE Great Lakes Bioenergy Research Center, University of Wisconsin—Madison, Wisconsin, USA*

*^b^Wisconsin Energy Institute, University of Wisconsin—Madison, Wisconsin, USA*

*^c^Laboratory of Genetics, University of Wisconsin—Madison, Wisconsin, USA*

*^d^Department of Civil and Environmental Engineering, University of Wisconsin–Madison, Wisconsin, USA*

*^e^Department of Bacteriology, University of Wisconsin–Madison, Wisconsin, USA*

*^‡^Benjamin Hall and Wayne Kontur contributed equally to this work. Author order was determined by order of increasing seniority.*

#Address correspondence to Timothy J. Donohue, [tdonohue@bact.wisc.edu](mailto:tdonohue@bact.wisc.edu).

*Present address: Jeanette C. Neri, Parexel, Newton, Massachusetts, USA. Derek M. Gille, Sword Bio, Chicago, Illinois, USA.

**Supplementary Information**

**Construction of plasmids for generating in-frame deletions of Saro_1814, Saro_1817, Saro_0236, or Saro_1168.** Regions of *Novosphingobium aromaticivorans* genomic DNA containing ~1000 bp upstream and downstream of the genes to be deleted were amplified via PCR (see Table S4 for primers). Plasmid pK18msB-MCS1 (a variant of pK18*mobsacB* (1) in which the multiple cloning site has been removed, and which contains a gene for kanamycin resistance and *sacB* for sucrose sensitivity) was linearized via PCR as previously described (2). The upstream and downstream flanking regions for each gene were combined with linearized pK18msB-MCS1 using the NEBuilder HiFi Assembly system (New England Biolabs, Ipswich, MA) to produce a plasmid in which the upstream and downstream DNA sequences are adjacent, with no intervening coding region (Table 1, main text). In all cases, a gene’s start codon was eliminated; for some genes, a portion of the downstream coding region was retained. The plasmids were transformed into NEB 5-alpha competent *Escherichia coli* cells (New England Biolabs). The transformed *E. coli* cells were cultured in LB media + kanamycin, the plasmids were purified using a Qiagen® Plasmid Maxi Kit (Qiagen, Germany), and DNA sequencing was used to confirm the presence of the desired junction between upstream and downstream fragments.

**Plasmids for recombining a foreign *crtW* gene into the *N. aromaticivorans* genome.** DNA fragments containing the gene coding for the CrtW protein from either *Sphingomonas taxi* ATCC 55669 (NCBI Accession WP_038660513.1) or *Sphingomonas astaxanthinifaciens* (NCBI accession WP_211248127.1), with sequences complementary to the upstream and downstream regions of Saro_0236 at the *crtW* fragments’ ends, were ordered as gBlocks from Integrated DNA Technologies (Coralville, IA). Genes encoding CrtW from other organisms were constructed to have codon usage frequencies similar to those of other genes in *N. aromaticivorans* (calculated from several genes in the genome), but without making the GC content of individual genes too high for the gBlock synthesis process. Sequences of the fragments were (with sequences complementary to the Saro_0236 flanks in lowercase):

*Sphingomonas taxi* ATCC 55669 *crtW*:

gtcaaccgcgtcaacctgttcaggcaggagccgttgggcATGAGCCCCGATCGGGGGAATACGCGCCACAGCCTGCTGCTCGCCGCCGCGATCGGTGCGGCCTGGCTCGCCATCCATATTGGCGGCATCTTCTTCTGGCAGTGGCGTGCCGCCACGGTGCCGGTCGCACTTCTGCTGATCGTGGTGCAGGCGTGGCTGAGCACCGGCCTCTTCATCGTCGCGCACGACTGCATGCACGGATCGTTCGCACCCGGACGCCGGGCGTGGAACGTCGTCGTCGGCACCCTGTGCCTCGGCGCCTATGCCGGCCTGTCCTATCGCGCGCTCTACCCGATGCACCACGCGCATCATGCCGCGCCCGGCACCGAACACGATCCCGACTTCCATGCCGCCGCGCCTCGCCGCGCGCTTCCGTGGTTCGTCCATTTCTTCCGCGGGTACTACACCCATGGCCAGATCCTGCGGATCACGCTTGCGGCGATCGTCTACATCCTGCTCGGCGCGTCGCTTCTCAACATCGTGCTGTTCTGGGCGGTGCCGGCGCTGCTCGCGCTTGCGCAATTGTTTCTGTTCGGCACCTATCTGCCCCACCGTCACGGCGAGACGCCGTTCGCCGACACGCACAACGCGCGCAGCAACTCGCTGTCGCCGCTCGCCTCGCTGGCGACCTGCTTCCACTTCGGTGCCTATCACCACGAACATCACCTCAGCCCGCAGACTCCGTGGTGGCAGCTCCCGCACATCAAGCGCGGCTGAcatcacgagcaataccgctgcaactatggcctctacttc

*Sphingomonas astaxanthinifaciens* *crtW*:

gtcaaccgcgtcaacctgttcaggcaggagccgttgggcATGGCAGAACGCCGTCGCCCGGCCTATATGGCACCCATGCTCAGTGATGCGCAGCGCCGTCGCCAGGCGATGATCGGCCTTGGCCTTGCCGCAGCGATCACCGCAGCCTTCGTCGCGCTTCATGTCTGGTCGGTCTTCTTCCTTCCGCTTGAAGGAGCAGGCTGGTGGCTTGCGCTTCCGATCGTCGCAGTGCAAACCTGGCTTAGCGTCGGTCTGTTCATCGTCGCGCATGATGCAATGCATGGCAGCCTTGCACCGGGCCGCCCTGCGACCAACCTTTTCTGGGGACGGCTTACGCTTCTGCTCTACGCGGGCTTCTGGTTGGACCGCCTTTCGCCCAAGCATTTCGACCACCACCGCCATGTCGGGACCGAGCGCGATCCCGATTTCTCGGTCGATCATCCGACCCGCTTCTGGCCCTGGTATTATGCCTTCATGCGGCGCTATTTCGGGCTTCGCGAATATCTGGTGCTGAACGCGCTGGTGCTGGCCTACGTGCTGGTGCTGAAGGCGCCGCTCGGCAATCTGCTCCTGTTCTGGGCGCTGCCCTCGATCCTGTCCTCGATCCAGCTCTTCTATTTCGGCACCTACCTTCCGCACCGGCACGAGGACGCGCCCTTCGCCGACCAGCACAATGCCCGCAGCAACGACTTTCCGGTCTGGCTGTCGCTGCTGACCTGCTTCCACTTCGGCTATCACCGCGAGCATCACCTCAGCCCCGGCACCCCGTGGTGGCAGCTGCCTCGACGACGGCGAGAGCTTGCACTTCCTGCATGAcatcacgagcaataccgctgcaactatggcctctacttc

The plasmid that was used to delete Saro_0236 was linearized using primers 5’-CCAACGGCTCCTGCCTGAACAG-3’ and 5’-CATCACGAGCAATACCGCTGCAACTATGG-3’. Each of the *crtW* DNA fragments was separately combined with this linearized plasmid using the NEBuilder HiFi Assembly system (New England Biolabs) to produce plasmids pK18msB/ΔSaro0236::StaxiW and pK18msB/ΔSaro0236::SastaW, each containing *crtW* in the Saro_0236 genomic locus (with the start and stop codons for *crtW* located where those codons for Saro_0236 would normally be located). The plasmids were transformed into NEB 5-alpha competent *E. coli* (New England Biolabs), and the plasmids were purified and confirmed as described above.

**Modifying the *N. aromaticivorans* genome.** The gene deletion and *crtW*-containing plasmids were separately mobilized into *N. aromaticivorans* via conjugation with *E. coli* S17-1. For conjugation, cultures of *E. coli* S17-1 harboring the plasmid (in LB containing kanamycin) and *N. aromaticivorans* (in GluSis) were grown overnight at 30 °C. Cultures were diluted and allowed to resume exponential growth before cells were harvested by centrifugation (~7,000 × *g* for 5 min). Each cell pellet was separately washed in LB, then resuspended together into 90 μL LB containing no added antibiotic. Conjugations were allowed to proceed overnight at 30 °C. The following day, the cells were harvested via centrifugation, resuspended into GluSis, and shaken at 200 rpm for >1 h at 30 °C. Cells from these cultures were plated onto solid GluSis with kanamycin to select for *N. aromaticivorans* cells in which the plasmid had integrated into the genome via homologous recombination (single crossovers). Single crossover strains were confirmed through the inability to immediately grow on GluSis containing 10% sucrose.

Single crossover strains were cultured in 5 mL of GluSis containing 10% sucrose and shaken at 30 °C until growth commenced (usually several days), which signified loss of the plasmid from the genome via a second round of homologous recombination. These cultures were streaked onto solid GluSis containing 10% sucrose to generate individual colonies that had lost the plasmid, and plasmid loss was confirmed by the inability to grow on GluSis containing kanamycin. The desired genomic modification was confirmed via PCR and sequencing of isolated genomic DNA.

**Measuring chemical oxygen demand (COD).** COD was measured on filtered and unfiltered samples using COD2 mercury-free high range (20–1,500 mg/L) digestion vials following the manufacturer’s protocol (2565115, Hach, Loveland, CO, United States). “Suspended solids” COD was calculated by subtracting the filtered sample COD from the unfiltered sample COD. “Uncharacterized” COD refers to the difference between the total filtered COD and the sum of all calculated CODs for every measured aromatic and organic molecule. The COD for each measured molecule was calculated based on the stoichiometry of how many moles of O_2_ would be required to fully oxidize the molecule to CO_2_ and H_2_O.

**Analysis of organics in sorghum APL.**

Analysis was performed on an Agilent 1260 Infinity II HPLC equipped with an HPX-87H column at 50°C and a refractive index detector. The mobile phase was 0.02N sulfuric acid flowing at 0.5 mL/min. Samples were centrifuged to remove cells and particulates, decanted, and diluted 1:9 (v/v) with MilliQ water. The sample injection volume was 50 µL. The analytes quantified were glucose, xylose, pyruvic acid, xylitol, cellobiose, succinic acid, lactic acid, propanoic acid, glycerol, formic acid, acetic acid, and ethanol. Concentrations were calculated from a 9-point calibration curve and are reported of the average of two technical replicates.

**Supplemental Figures and Tables**

**Figure S1.** HPLC analysis and absorbance spectra of carotenoid and CoQ_10_ standards used in this study. Data are shown in milliabsorbance units (mAu).


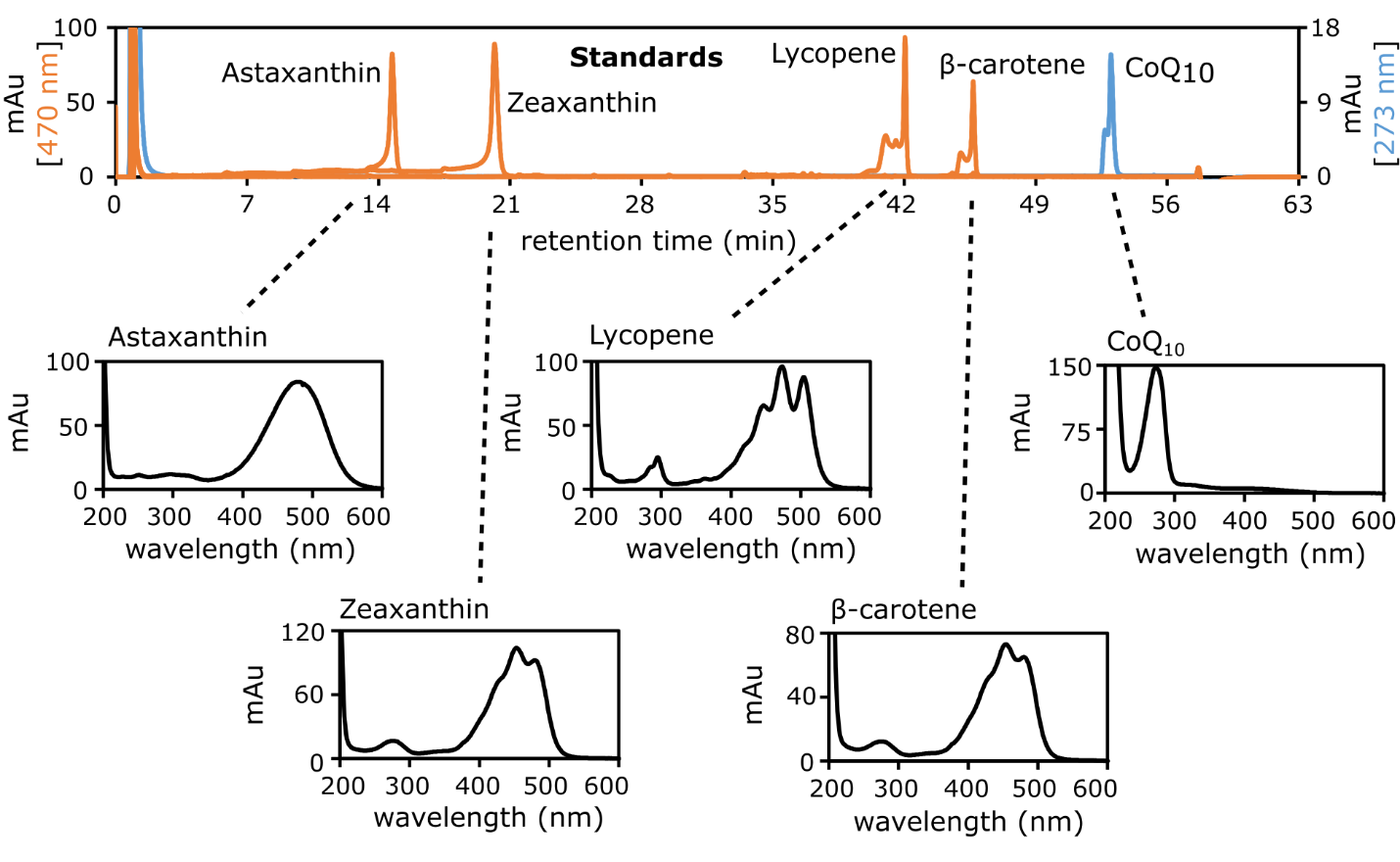


**Figure S2.** Compounds detected in HPLC analyses of *N. aromaticivorans* 12444Δ1879 acetone:methanol extracts. HPLC data reproduced from Figure 2 of the main text (A) is used to organize the absorbance spectra of different compounds (B), some of which have associated mass spectrometry scans (C). Dotted lines in (A) denote retention times of carotenoid standards.


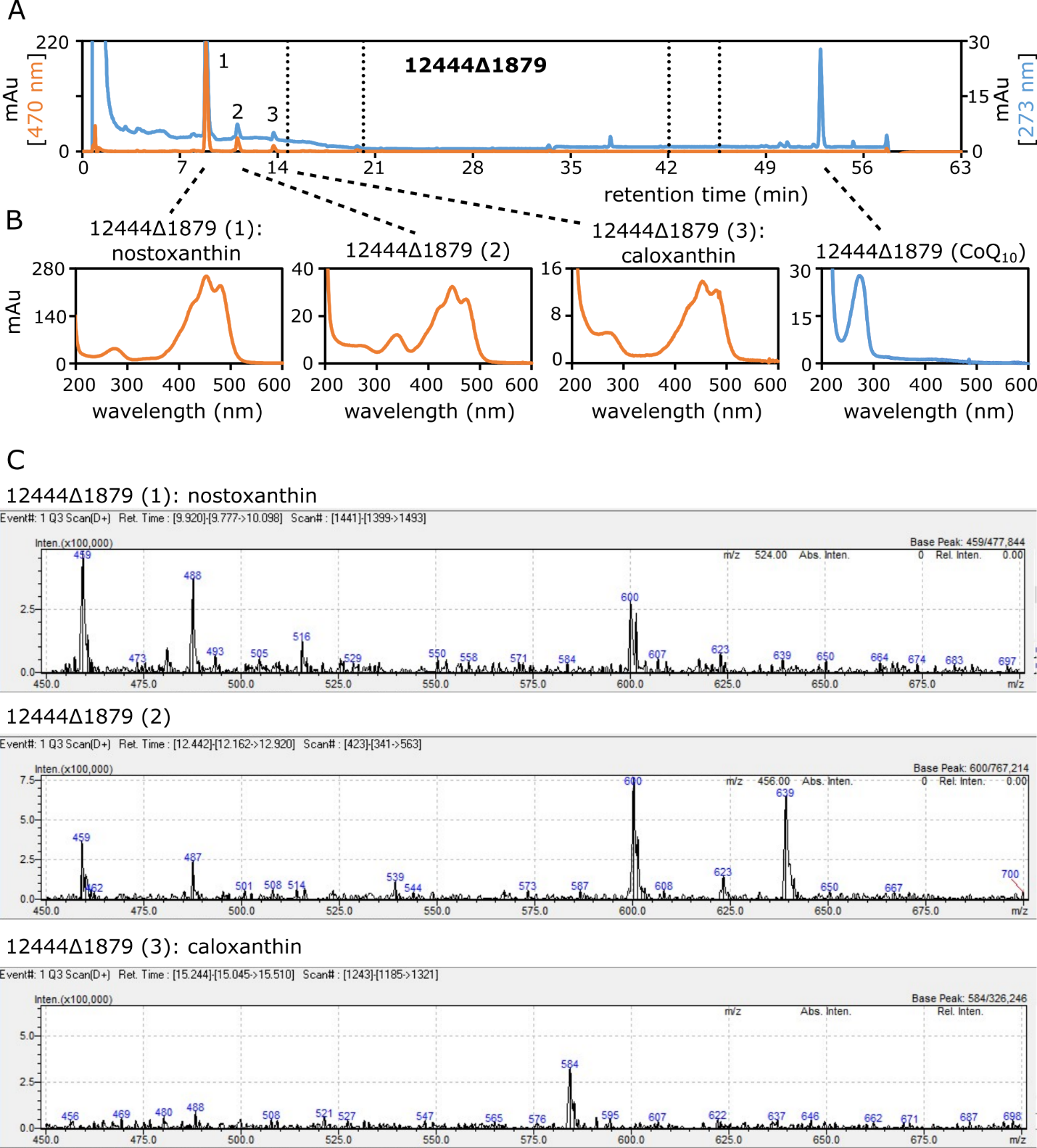


**Figure S3.** Compounds detected in HPLC analyses of *N. aromaticivorans* 12444ΔcrtB acetone:methanol extracts. HPLC data reproduced from Figure 2 of the main text (A) is used to organize the absorbance spectra of different compounds (B).


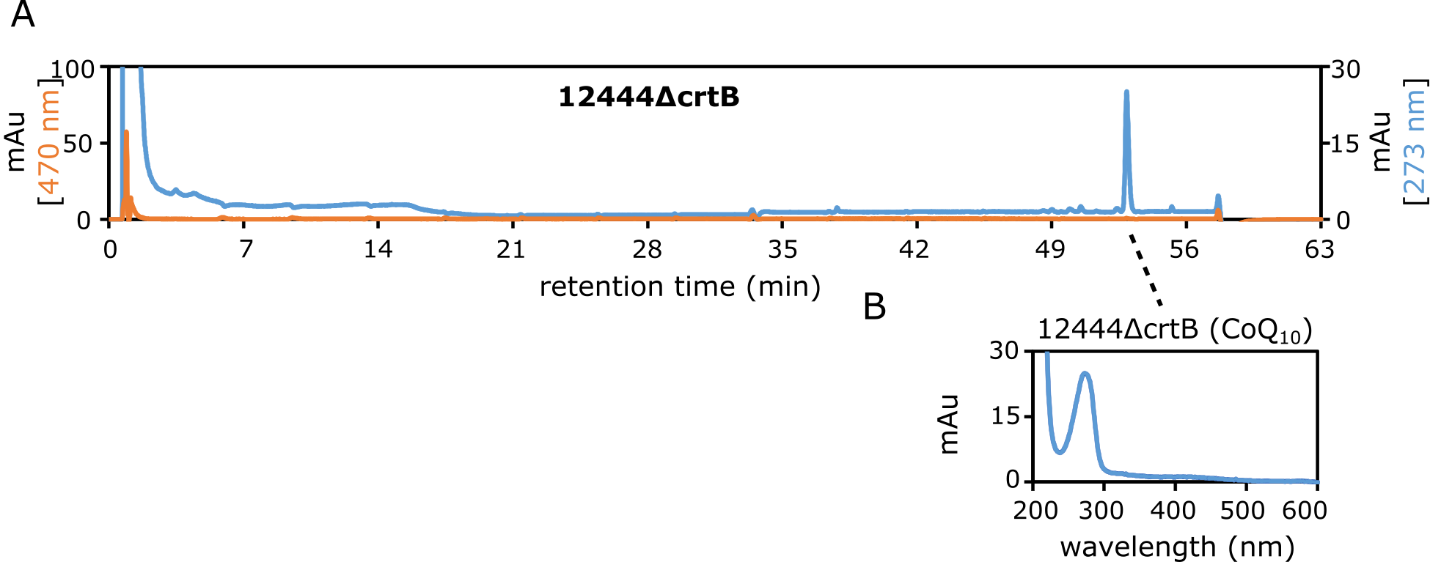


**Figure S4.** Compounds detected in HPLC analyses of *N. aromaticivorans* 12444ΔcrtY acetone:methanol extracts. HPLC data reproduced from Figure 2 of the main text (A) is used to organize the absorbance spectra of different compounds (B). Dotted lines in (A) denote retention times of carotenoid standards.


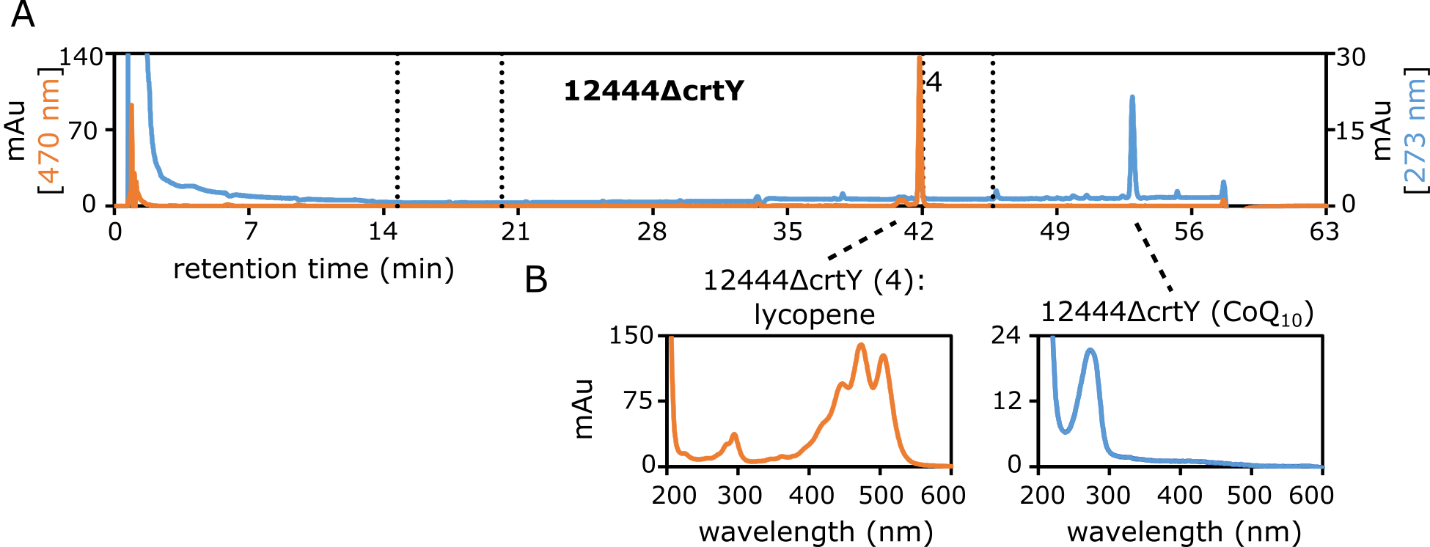


**Figure S5.** Compounds detected in HPLC analyses of *N. aromaticivorans* 12444ΔcrtG acetone:methanol extracts. HPLC data reproduced from Figure 2 of the main text (A) is used to organize the absorbance spectra of different compounds (B). Dotted lines in (A) denote retention times of carotenoid standards.


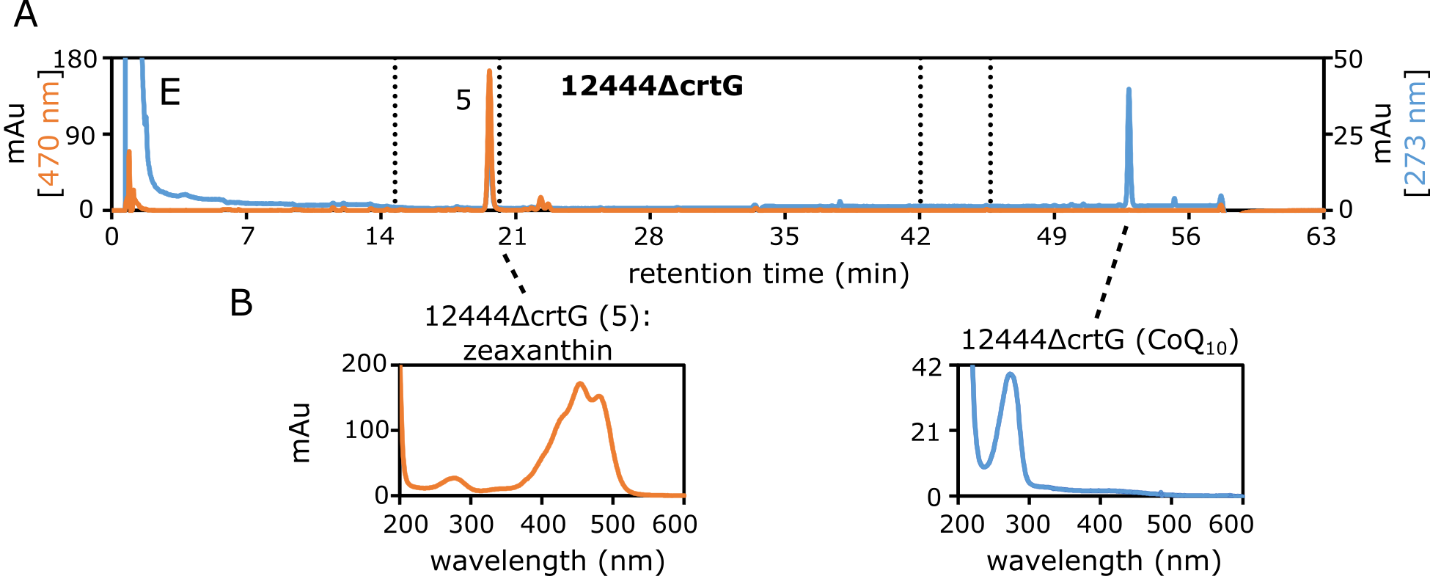


**Figure S6.** Compounds detected in HPLC analyses of *N. aromaticivorans* 12444ΔcrtGZ acetone:methanol extracts. HPLC data reproduced from Figure 2 of the main text (A) is used to organize the absorbance spectra of different compounds (B). Dotted lines in (A) denote retention times of carotenoid standards.


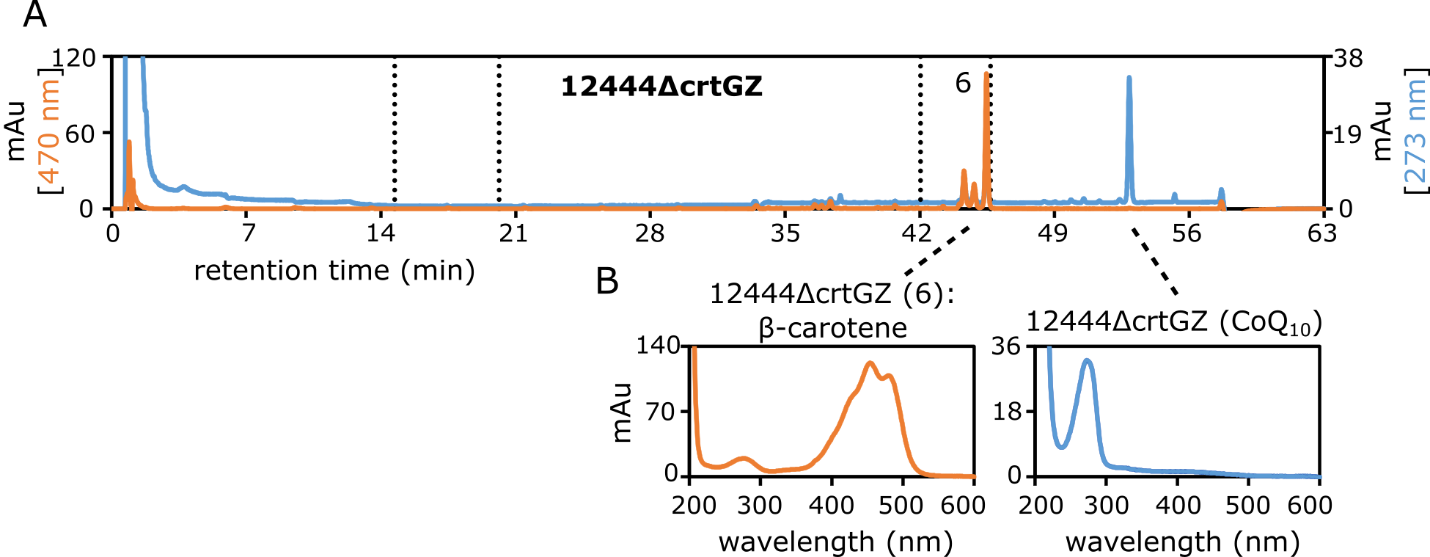


**Figure S7.** Compounds detected in HPLC analyses of *N. aromaticivorans* 12444SastaW acetone:methanol extracts. HPLC data reproduced from Figure 2 of the main text (A) is used to organize the absorbance spectra of different compounds (B), some of which have associated mass spectrometry scans (C). Compound 8’s absorbance was too low to obtain an accurate absorbance or mass spectrum. Dotted lines in (A) denote retention times of carotenoid standards.


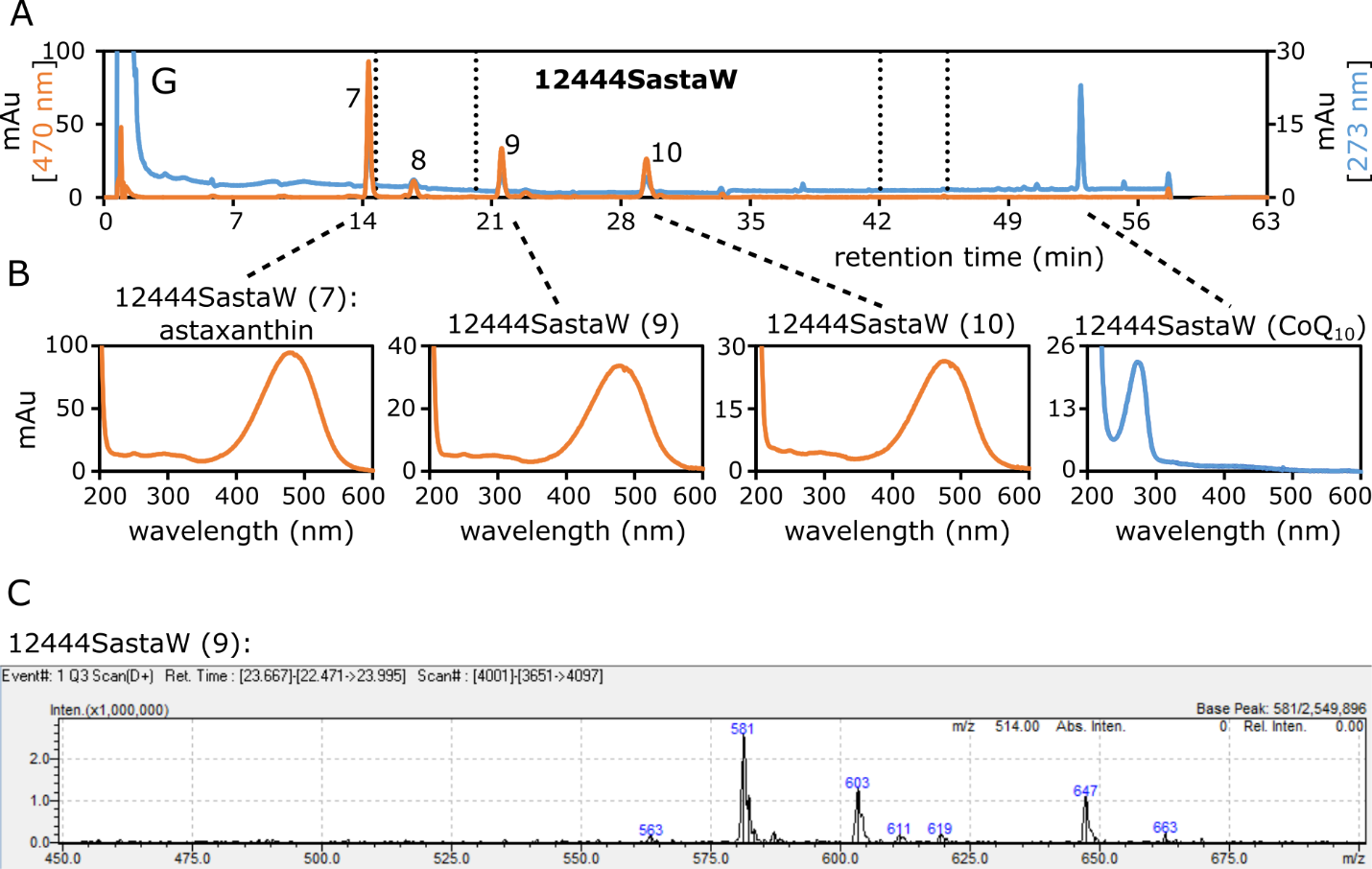


**Figure S8.** Compounds detected in HPLC analyses of *N. aromaticivorans* 12444StaxiW acetone:methanol extracts. HPLC data reproduced from Figure 2 of the main text (A) is used to organize the absorbance spectra of different compounds (B), some of which have associated mass spectrometry scans (C). Dotted lines in (A) denote retention times of carotenoid standards.


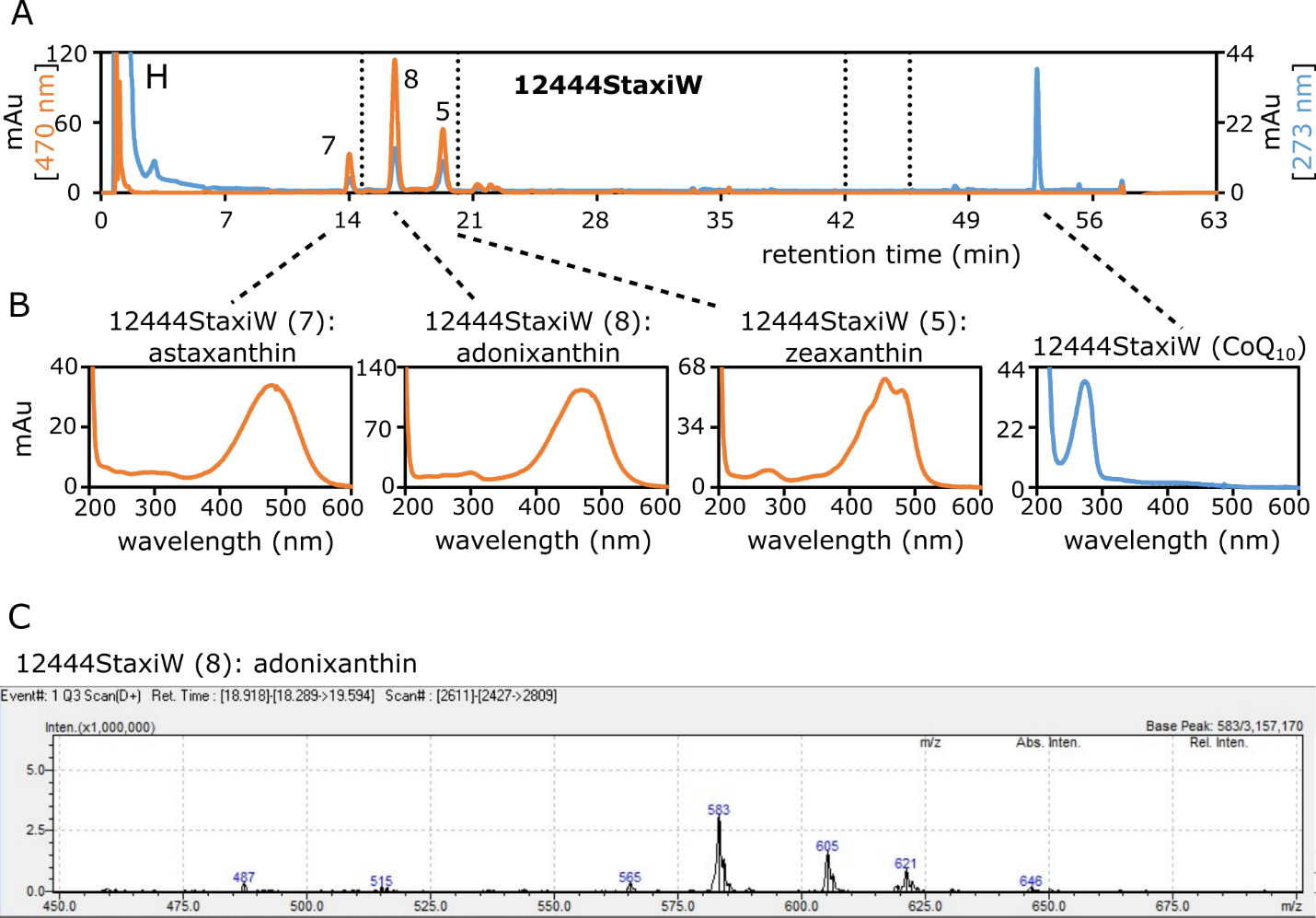


**Figure S9.** Chemical oxygen demand (COD) of sorghum APL before and after incubation with indicated *N. aromaticivorans* strains (manuscript Figure 5). All detected compounds in Table S2 are included in the graphs, but many are too low in abundance to be visible in the figure. COD provides an estimate of the available chemical energy in a solution by measuring how much oxygen is required to fully oxidize the organic molecules to CO_2_. COD was measured as described in Supplementary Information.


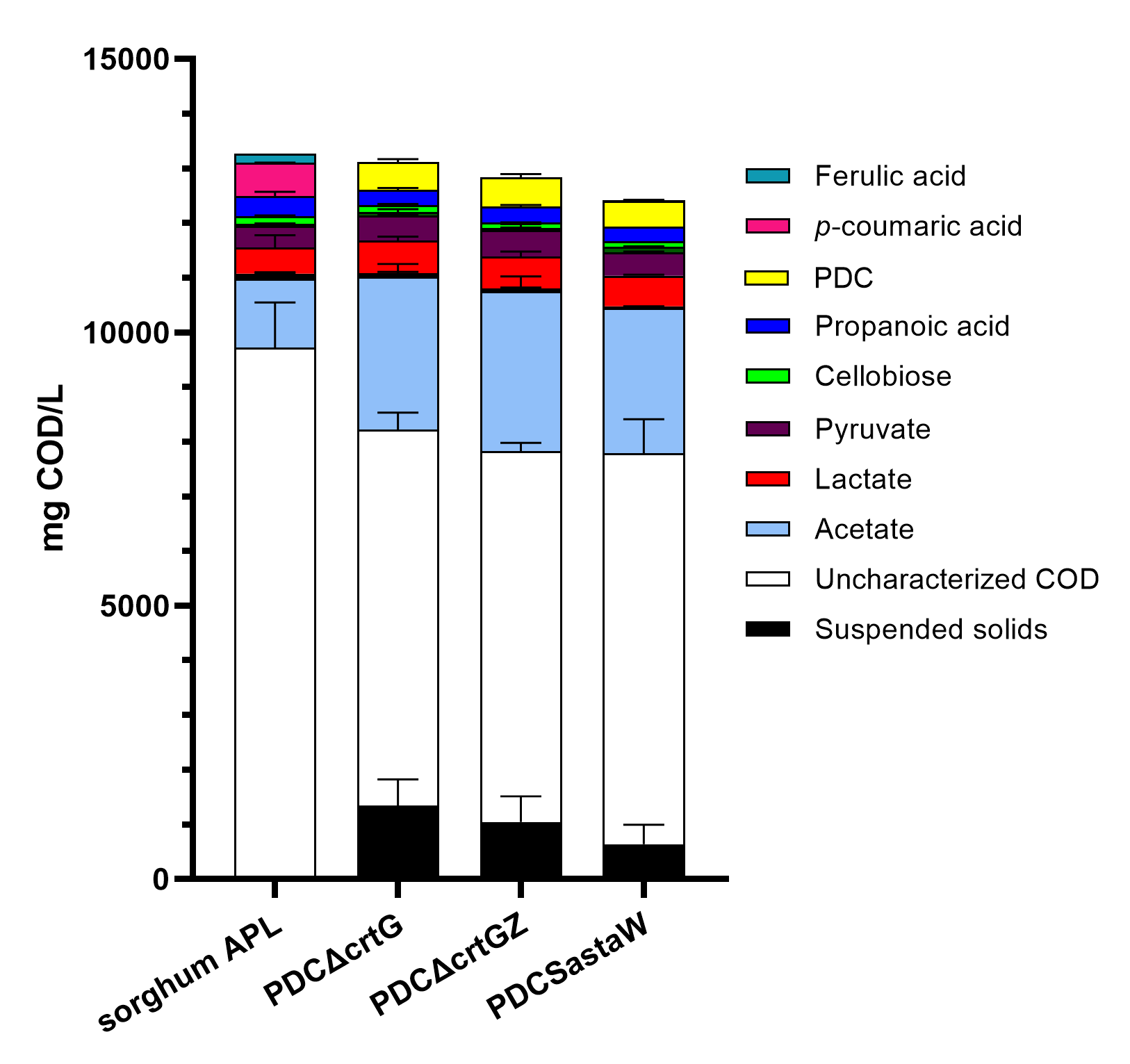


**Figure S10.** Dry cell weights normalized to 10 mL of culture of *N. aromaticivorans* strains grown in SMB + 4 mM vanillate at different O_2_ concentrations (A) or in sorghum APL in shake flasks (B).


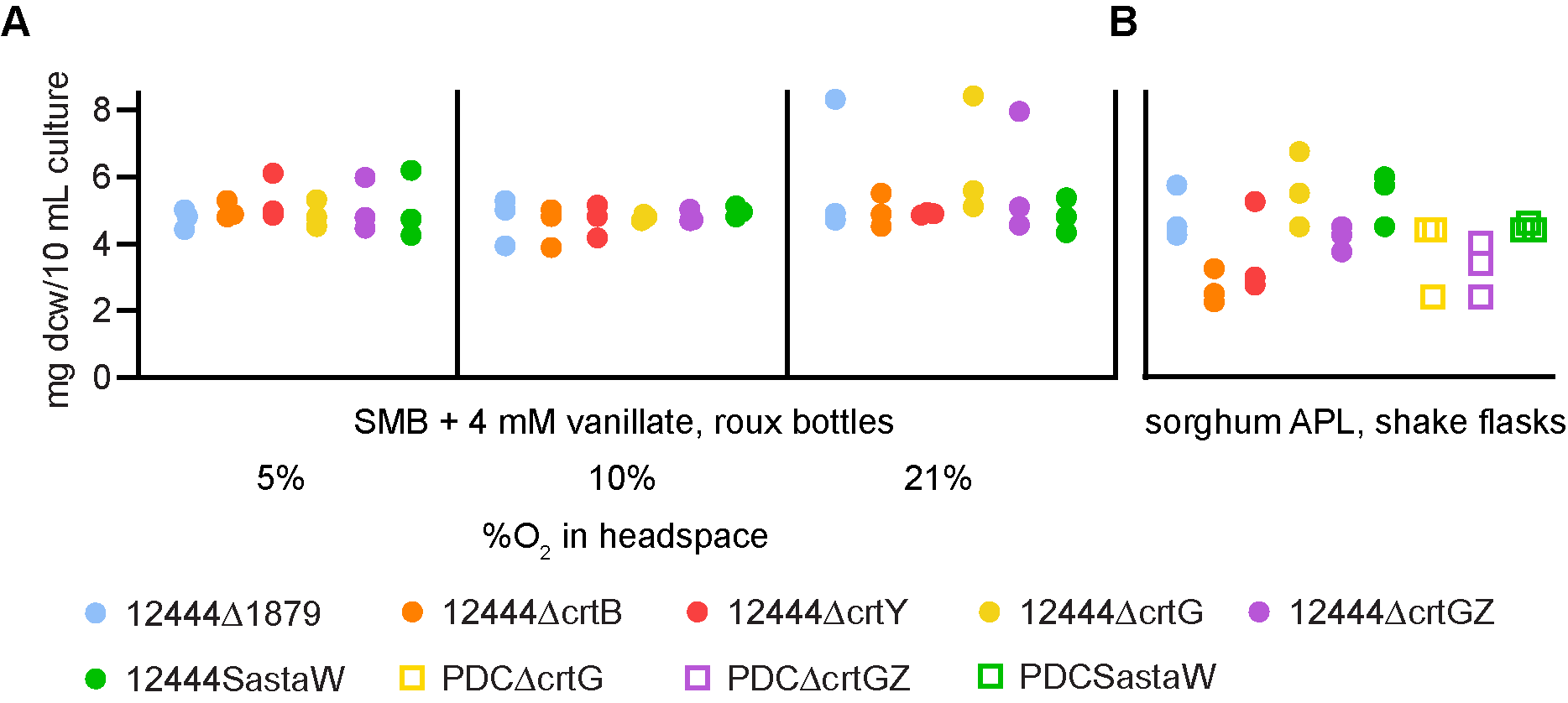


**Figure S11.** Mobile phase binary gradient for HPLC analysis of compounds in *N. aromaticivorans* acetone:methanol extracts. Solvent A is 70% acetonitrile/30% water and Solvent B is 70% acetonitrile/30% isopropanol. The method proceeds for 63 min.


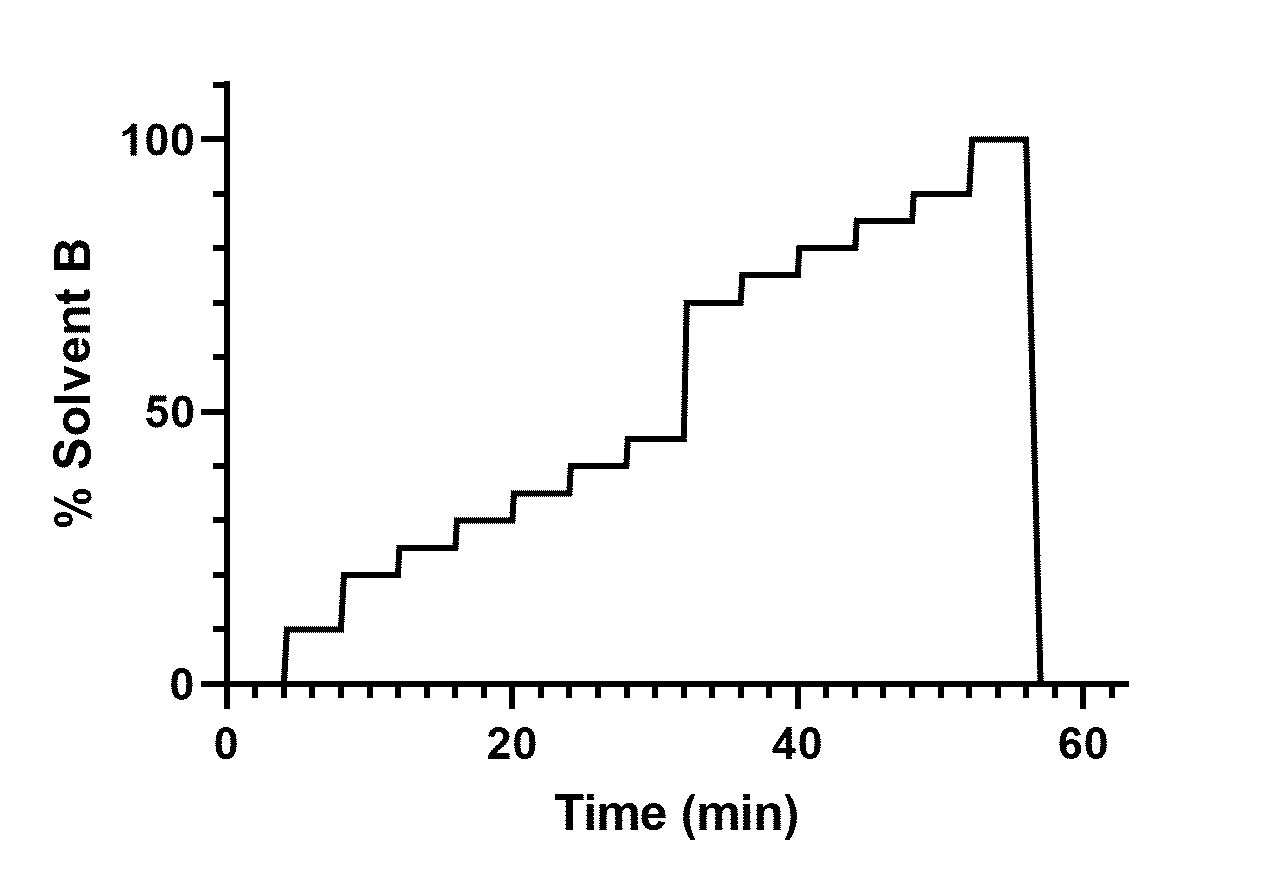


**Figure S12.** Mobile phase binary gradient for HPLC analysis of aromatic compounds in sorghum APL. Solvent A is 0.2% formic acid in water and solvent B is methanol. The method ends at 10 min.

**
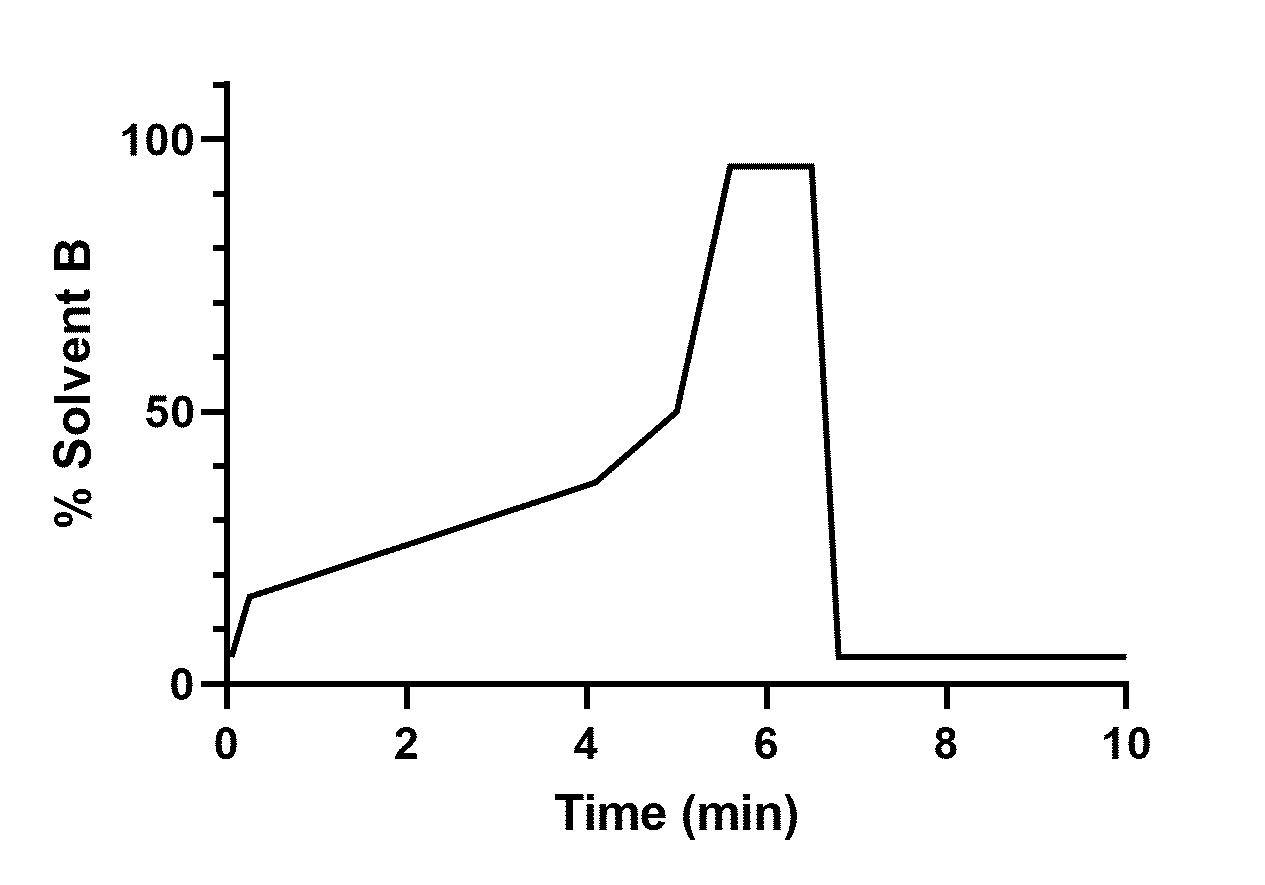
**

**Table S1.** Amino acid sequence identity of predicted *N. aromaticivorans* DSM 12444 carotenoid pathway proteins with the most closely related characterized protein identified by PaperBLAST (3).

| **Protein annotation** | **Locus tag, accession number** | **% identity** | **Most similar protein accession number** | **Organism encoding most similar protein** | **Reference for characterization** |
| --- | --- | --- | --- | --- | --- |
| CrtI | Saro_1816, [ABD26256.1](https://www.ncbi.nlm.nih.gov/protein/ABD26256.1/) | 74% | [ADO33738.1](https://www.ncbi.nlm.nih.gov/protein/ADO33738.1) | *Sphingomonas elodea* ATCC 31461 | (4) |
| CrtY | Saro_1817, [ABD26257.1](https://www.ncbi.nlm.nih.gov/protein/ABD26257.1) | 52% | [AEP37353.1](https://www.ncbi.nlm.nih.gov/protein/AEP37353.1) | *S. elodea* ATCC 31461 | (4) |
| CrtZ | Saro_1168, [ABD25613.1](https://www.ncbi.nlm.nih.gov/protein/ABD25613.1) | 58% | [AIT05942.1](https://www.ncbi.nlm.nih.gov/protein/AIT05942.1) | *Sphingomonas taxi* ATCC 55669 | (5) |
| CrtG | Saro_0236, [WP_011443898.1](https://www.ncbi.nlm.nih.gov/protein/WP_011443898.1) | 72% | [AEP37351.1](https://www.ncbi.nlm.nih.gov/protein/AEP37351.1) | *S. elodea* ATCC 31461 | (4) |

**Table S2.** Concentration of aromatic monomers and other organics in sorghum APL used in this study. Values represent mean ± S.D.. Trace levels of indicated aromatic compounds were detected, but concentrations << 0.1 mM prevented quantification. All aromatics in Table S3 were investigated. Other organics were measured as described in Supplementary Information. ND, not detected.

| **Compound** | **Concentration (mM)** | | | |
| --- | --- | --- | --- | --- |
|  | Sorghum APL (n = 2) | PDCΔcrtG (n = 3) | PDCΔcrtGZ (n = 3) | PDCSastaW (n = 3) |
| **Aromatic monomers** |  |  |  |  |
| *p*-coumaric acid | 2.01 ± 0.03 | ND | ND | ND |
| Ferulic acid | 0.513 ± 0.001 | ND | ND | ND |
| PDC | ND | 3.2 ± 0.3 | 3.3 ± 0.4 | 2.9 ± 0.1 |
| Acetosyringone | Trace | ND | ND | ND |
| *p*-hydroxybenzaldehyde | Trace | ND | ND | ND |
| *p*-hydroxybenzoic acid | Trace | ND | ND | ND |
| Protocatechuic acid | Trace | ND | ND | ND |
| Vanillin | Trace | ND | ND | ND |
| Syringic acid | ND | Trace | ND | Trace |
| Vanillic acid | ND | Trace | ND | Trace |
| **Other organics** |  |  |  |  |
| Acetate | 19.8 ± 0.9 | 43 ± 4 | 45 ± 4 | 41.5 ± 0.3 |
| Lactate | 5 ± 2 | 6.3 ± 0.7 | 6.2 ± 0.9 | 5.8 ± 0.2 |
| Pyruvate | 4.8 ± 0.3 | 5.7 ± 0.3 | 6.1 ± 0.5 | 5.4 ± 0.2 |
| Propanoic acid | 3.2 ± 0.7 | 2.5 ± 0.3 | 2.6 ± 0.2 | 2.44 ± 0.02 |
| Formate | 2.8 ± 0.7 | 2.0 ± 0.2 | 0.98 ± 0.08 | 1.5 ± 0.2 |
| Cellobiose | 0.36 ± 0.03 | 0.32 ± 0.07 | 0.247 ± 0.005 | 0.260 ± 0.006 |
| Xylitol | 0.25 ± 0.06 | 0.4 ± 0.3 | 0.2 ± 0.4 | 0.54 ± 0.06 |
| Glucose | 0.1 ± 0.1 | 0.1 ± 0.1 | 0.1 ± 0.1 | ND |
| Ethanol | 0.03 ± 0.02 | 0.2 ± 0.1 | 0.09 ± 0.08 | 0.08 ± 0.09 |
| Glycerol | 0.2 ± 0.2 | ND | ND | ND |
| Succinate | ND | ND | ND | ND |
| Xylose | ND | ND | ND | ND |

**Table S3.** Multiple reaction module conditions for HPLC-MS/MS identification of aromatic compounds in sorghum APL.

| **Compound** | **MW  (g/ mol)** | **DUIS mode** | **Parent m/z** | **Transition 1 m/z, collision energy (V)** | **Transition 2 m/z, collision energy (V)** | **Transition 3 m/z, collision energy (V)** |
| --- | --- | --- | --- | --- | --- | --- |
| Acetosyringone | 196.2 | + | 197.0 | 155.1, (-20) | 140.1, (-28) | 125.1, (-32) |
| Acetovanillone | 166.2 | + | 167.0 | 125.1, (-13) | 110.0, (-23) | N/A |
| Catechol | 110.1 | - | 109.2 | 91.1, (24) | 65.1, (24) | 41.0, (35) |
| Ferulic acid | 194.2 | - | 193.0 | 134.1, (17) | 149.1, (16) | 178.1, (14) |
| p-Coumaric acid | 164.0 | - | 163.0 | 119.1, (16) | 93.1, (31) | 117.1, (30) |
| PDC | 184.1 | - | 183.1 | 139.0, (12) | 111, (15) | 95.1, (13) |
| p-OH-Benzaldehyde | 122.1 | - | 121.0 | 92.1, (25) | 93.1, (21) | 65.1, (24) |
| p-OH-benzoic acid | 138.1 | - | 137.0 | 93.0, (16) | 65.1, (33) | 75.2, (30) |
| Protocatechuic acid | 154.1 | - | 153.0 | 109.1, (17) | 91.1, (26) | N/A |
| Syringaldehyde | 182.2 | - | 181.1 | 151.1, (26) | 166.2, (20) | 123.1, (27) |
| Syringic acid | 198.2 | - | 197.0 | 182.1, (14) | 122.8, (23) | 94.9, (33) |
| Vanillic acid | 168.1 | - | 167.0 | 152.1, (19) | 107.9, (19) | 123.0, (14) |
| Vanillin | 152.2 | - | 151.0 | 136.0, (17) | 92.0, (22) | 108.0, (24) |

**Table S4.** Photodiode array detection parameters for quantification of aromatic compounds in sorghum APL.

| **Compound** | **Peak absorbance wavelength (nm)** | **Wavelength used for quantification (nm)** | **Retention time (min)** |
| --- | --- | --- | --- |
| Ferulic acid | 322 | 325 | 3.9 |
| *p*-Coumaric acid | 312 | 334 | 3.3 |
| PDC | 315 | 325 | 1.2 |
| Protocatechuic acid | 259 | 258 | 1.8 |
| Vanillic acid | 260 | 258 | 2.7 |

**Table S5.** Primers used to create cloning vectors for genomic modifications of *N. aromaticivorans.*

| **Gene deleted / plasmid linearized** | **Fragment (relative to gene)** | **Primers** |
| --- | --- | --- |
| *crtB* (Saro_1814) | Upstream | 5’-CGATTCATTAATGCAGCTGGCACGACAGCAGGACTCTCGATCTACCTGCA  CCATC-3’  5’-CGATAAAGCCCAGCTTGCTCACAGGTCGTCGGCCTTCATTGC-3’ |
|  | Downstream | 5’-GAAGGCCGACGACCTGTGAGCAAGCTGGGCTTTATCGGCAAAGC-3’  5’-GTTTCTGCGGACTGGCTTTCTAGATGTTCCACCATGACGAGGTGGACCAG  AATGAAC-3’ |
| *crtY* (Saro_1817) | Upstream | 5’-CGATTCATTAATGCAGCTGGCACGACAGCTTGAAACGGTAGCCGAAGGT  GTAAAGGTCG-3’  5’-GCAAATGAAAGTGGGTTGGCGATCCGCTTAGGGACATGCGGTTG-3’ |
|  | Downstream | 5’-CATGTCCCTAAGCGGATCGCCAACCCACTTTCATTTGCAGGAACC-3’  5’-GTTTCTGCGGACTGGCTTTCTAGATGTTCGATGGTGCAGGTAGATCGAGA  AGTCCTG-3’ |
| *crtG* (Saro_0236) | Upstream | 5’-CGATTCATTAATGCAGCTGGCACGACAGGTCGAACAGTACGTCACCTTCA  TCAACCAG-3’  5’-CGGTATTGCTCGTGATGCCAACGGCTCCTGCCTGAACAG-3’ |
|  | Downstream | 5’-GCAGGAGCCGTTGGCATCACGAGCAATACCGCTGCAACTATGG-3’  5’-GTTTCTGCGGACTGGCTTTCTAGATGTTCCTCGTATCCCACAGCGATATC  AGGATGC-3’ |
| *crtZ* (Saro_1168) | Upstream | 5’-CGATTCATTAATGCAGCTGGCACGACAGCACTTCCATCGTCTTCGACTGC  TTGAG-3’  5’-CCTGCTTCAGCACCGCAGCGACACTTTCTTACAATTTGCCCGAAAGTC-3’ |
|  | Downstream | 5’-GGCAAATTGTAAGAAAGTGTCGCTGCGGTGCTGAAGCAGGAACTG-3’  5’-GTTTCTGCGGACTGGCTTTCTAGATGTTCCTACTGCCGGATTTTCCGGCAT  GGAAG-3’ |
| pK18msB-MCS1 |  | 5’-CTGTCGTGCCAGCTGCATTAATG-3’  5’-GAACATCTAGAAAGCCAGTCCGCAGAAAC-3’ |
| pK18msB/ΔSaro0236 |  | 5’-CCAACGGCTCCTGCCTGAACAG-3’  5’-CATCACGAGCAATACCGCTGCAACTATGG-3’ |

Underlined sequences are extensions to the primers that will not bind to the genomic DNA region being amplified. Blue and green sequences are complementary to the end regions of linearized pK18msB-MCS1. The red and purple sequences are complementary to the red and purple sequences of the primer used to create the other flanking region for the same gene.

**Table S6.** Carotenoid yields of *N. aromaticivorans* strains grown in sorghum APL medium, normalized by the mass of sorghum used to make APL.

| Strain | Carotenoid yield (μg/g sorghum) | CoQ_10_ yield (μg/g sorghum) |
| --- | --- | --- |
| 12444Δ1879 | HPLC peak area: 6.1 ± 0.6×10^8^/g sorghum (Nostoxanthin) | 1.9 ± 0.2 |
| 12444ΔcrtB | None detected | 1.9 ± 0.4 |
| 12444ΔcrtY | 2.0 ± 0.6 (Lycopene) | 1.8 ± 0.2 |
| 12444ΔcrtG | 3.0 ± 0.4 (Zeaxanthin) | 1.5 ± 0.9 |
| 12444ΔcrtGZ | 3.7 ± 0.7 (β-carotene) | 1.8 ± 0.3 |
| 12444SastaW | 0.7 ± 0.2 (Astaxanthin) | 1.4 ± 0.3 |
| PDCΔcrtG | 2.9 ± 0.7 (Zeaxanthin) | 1.7 ± 0.2 |
| PDCΔcrtGZ | 2.0 ± 0.2 (β-carotene) | 1.4 ± 0.1 |
| PDCSastaW | 1.24 ± 0.06 (Astaxanthin) | 1.9 ± 0.1 |

**References**

1. Schäfer, A., A. Tauch, W. Jäger, J. Kalinowski, G. Thierbach, and A. Pühler. 1994. Small mobilizable multi-purpose cloning vectors derived from the *Escherichia coli* plasmids pK18 and pK19: selection of defined deletions in the chromosome of *Corynebacterium glutamicum*. *Gene*. 145:69–73.

2. Kontur, W.S., C.A. Bingman, C.N. Olmsted, D.R. Wassarman, A. Ulbrich, D.L. Gall, R.W. Smith, L.M. Yusko, B.G. Fox, D.R. Noguera, J.J. Coon, and T.J. Donohue. 2018. *Novosphingobium aromaticivorans* uses a Nu-class glutathione S-transferase as a glutathione lyase in breaking the β-aryl ether bond of lignin. *J. Biol. Chem.* 293:4955–4968.

3. Price Morgan N. and Arkin Adam P. 2017. PaperBLAST: Text Mining Papers for Information about Homologs. *mSystems*. 2:10.1128/msystems.00039-17.

4. Zhu, L., X. Wu, O. Li, C. Qian, and H. Gao. 2012. Cloning and characterization of genes involved in nostoxanthin biosynthesis of *Sphingomonas elodea* ATCC 31461. *PLoS One*. 7:e35099.

5. Ma, T., Y. Zhou, X. Li, F. Zhu, Y. Cheng, Y. Liu, Z. Deng, and T. Liu. 2016. Genome mining of astaxanthin biosynthetic genes from *Sphingomonas* sp. ATCC 55669 for heterologous overproduction in *Escherichia coli*. *Biotechnol J*. 11:228–237.
